# Transcriptomic and Phenotypic Analyses of the Sigma B-Dependent Characteristics and the Synergism between Sigma B and Sigma L in *Listeria monocytogenes* EGD-e

**DOI:** 10.3390/microorganisms8111644

**Published:** 2020-10-23

**Authors:** Mirjami Mattila, Panu Somervuo, Hannu Korkeala, Roger Stephan, Taurai Tasara

**Affiliations:** 1 Department of Food Hygiene and Environmental Health, Faculty of Veterinary Medicine, University of Helsinki, P.O. Box 66, 00014 Helsinki, Finland; mirjami.mattila@gmail.com (M.M.); panu.somervuo@helsinki.fi (P.S.); hannu.korkeala@helsinki.fi (H.K.); 2 Institute for Food Safety and Hygiene, Vetsuisse Faculty, University of Zurich, Winterthurerstr. 272, CH-8057 Zurich, Switzerland; stephanr@fsafety.uzh.ch

**Keywords:** *Listeria monocytogenes*, Sigma B, Sigma L, stress adaptation, cold stress, gene expression, transcription regulation, organic acid stress, ethanol stress

## Abstract

Numerous gene expression and stress adaptation responses in *L. monocytogenes* are regulated through alternative sigma factors σ^B^ and σ^L^. Stress response phenotypes and transcriptomes were compared between *L. monocytogenes* EGD-e and its Δ*sigB* and Δ*sigBL* mutants. Targeted growth phenotypic analysis revealed that the *ΔsigB* and *ΔsigBL* mutants are impaired during growth under cold and organic-acid stress conditions. Phenotypic microarrays revealed increased sensitivity in both mutants to various antimicrobial compounds. Genes de-regulated in these two mutants were identified by genome-wide transcriptome analysis during exponential growth in BHI. The Δ*sigB* and Δ*sigBL* strains repressed 198 and 254 genes, respectively, compared to the parent EGD-e strain at 3 °C, whereas 86 and 139 genes, respectively, were repressed in these mutants during growth at 37 °C. Genes repressed in these mutants are involved in various cellular functions including transcription regulation, energy metabolism and nutrient transport functions, and viral-associated processes. Exposure to cold stress induced a significant increase in σ^B^ and σ^L^ co-dependent genes of *L. monocytogenes* EGD-e since most (62%) of the down-regulated genes uncovered at 3 °C were detected in the Δ*sigBL* double-deletion mutant but not in Δ*sigB* or Δ*sigL* single-deletion mutants. Overall, the current study provides an expanded insight into σ^B^ and σ^L^ phenotypic roles and functional interactions in *L. monocytogenes*. Besides previously known σ^B^- and σ^L^-dependent genes, the transcriptomes defined in Δ*sigB* and Δ*sigBL* mutants reveal several new genes that are positively regulated by σ^B^ alone, as well as those co-regulated through σ^B^- and σ^L^-dependent mechanisms during *L. monocytogenes* growth under optimal and cold-stress temperature conditions.

## 1. Introduction

The Gram-positive, facultatively intracellular food-borne pathogen *Listeria monocytogenes* is the causative agent of serious food-borne illness, listeriosis. *L. monocytogenes* can survive and proliferate at refrigeration temperatures and also under alkaline, acid, and osmotic stresses [1,2,3]. Since *L. monocytogenes* is widespread in the environment and can be found in soil, water, and decaying vegetation, it can easily access raw materials used by the food industry causing a significant microbial control challenge in food-processing facilities [4,5,6,7,8,9,10,11].

Food-borne pathogens need to possess various types of strategies at the molecular level to tolerate different environmental conditions in foods and also inside the host. Transcriptional regulation of genes essential for growth under stress conditions that the bacteria have encountered, is among the most central mechanisms. Bacteria can regulate the transcription of genes through alternative sigma factors which control the function and promoter selectivity of bacterial RNA polymerase. *L. monocytogenes* has four known alternative sigma factors: σ^B^, σ^C^, σ^H^, and σ^L^. The primary sigma factor σ^D^ is involved in housekeeping gene expression control. In *L. monocytogenes*, sigma-70 family comprises σ^B^, σ^C^, σ^D^, and σ^H^, while σ^L^ is the only member in the sigma-54 family [12]. σ^B^ is the most extensively characterized sigma factor and is regarded as the major stress response regulator of *L. monocytogenes,* while in many other pathogenic bacteria, σ^L^ (*rpoN*) is considered central to regulation of the bacterial cell exterior [13].

The general stress responsive σ^B^ has been shown to play a role in adaptation to diverse stress conditions and in virulence responses of *L. monocytogenes*. Previous studies have illustrated σ^B^ to be part of response mechanisms involved in tolerance of osmotic, acid, alkaline, oxidative, extreme temperature, and bile salt stresses [14,15,16,17,18,19,20,21]. σ^B^ contributes to virulence by co-regulating genes that are critical for *L. monocytogenes* entry into host cells with the positive regulatory factor A protein (PrfA) [22,23,24]. Studies that have explored σ^B^-mediated gene expression and protein production in *L. monocytogenes* in different environmental conditions have shown that this alternative sigma factor has a large and diverse regulon [17,18,25,26,27,28,29,30,31,32]. Although a great number of genome-wide expression analysis studies have been made using *L. monocytogenes sigB* null mutant, a whole genome transcription analysis of *L. monocytogenes sigB* null mutant at low growth temperature has not been reported to date.

σ^L^ has not been as extensively characterized as σ^B^ in *L. monocytogenes*, although it has been shown to be involved in *L. monocytogenes* salt, acid, ethanol, and low temperature stress tolerance [33,34,35,36]. Liu et al. [37] were the first to detect elevated *rpoN* transcript levels in bacteria grown at 10 °C compared with 37 °C, whereas Arous et al. [38] were the first to study the σ^L^ regulon using a global gene-expression analysis and a *rpoN* mutant of *L. monocytogenes*. Genes regulated by σ^L^ at different growth temperatures were further studied in our previous work [36]. Genome-wide expression analysis revealed 237 and 203 genes as positively regulated by σ^L^ at 3 and 37 °C, respectively.

Previous studies [28,29] proposed considerable overlap between σ^B^ and σ^L^ regulons. Nevertheless, both of the studies were conducted at 37 °C. Consequently, the possible regulon overlaps between σ^B^ and σ^L^ at low growth temperature still remain undescribed.

In this study, we employed DNA-microarray based whole-genome expression analysis to investigate the role of σB in the growth of *L. monocytogenes* at 3 and 37 °C and identified the components of the σB regulon that participate in cold stress tolerance of *L. monocytogenes*. We also identified differentially expressed genes at 3 and 37 °C in *L. monocytogenes* EGD-e wild-type strain and its Δ*sigBL* mutant. Furthermore, we evaluated the possible regulon overlaps and synergism between σB and σL at low growth temperature using deletion mutant strains *ΔsigB* and *ΔsigBL*. Phenotypic microarrays, growth-curve analysis under different stress conditions, motility assays, and electron microscopy were used for the phenotypic characterization of the wild-type strain and its null mutants at 3 and 37 °C.

## 2. Materials and Methods

### 2.1. Bacterial Strains and Growth Conditions

Bacterial strains used in this study are listed in Table S1. Strains were grown on blood agar plates (bioTRADING, Mijdrecht, The Netherlands; Difco Laboratories, Detroit, MI, USA), in brain heart infusion (BHI) broth (Difco Laboratories) or defined minimal medium (DM) [39]. Growth media for testing the stress tolerance of the strains were generated as previously described [36]. Briefly, acid stress was generated by supplementation of 2.5% of lactic acid, 0.35% of acetic acid, or 0.35% of citric acid to normal BHI, and adjusting pH values to 6.0, 5.5, and 5.5, respectively [40]. Ethanol stress was generated by addition of 100% ethanol to a final concentration of 5% (*v/v*) into DM. Inocula were prepared from frozen stocks by streaking the strains onto blood agar and incubating plates overnight at 37 °C. Single colonies were inoculated into 10 mL BHI broth and grown to stationary growth phase (approximately 10^9^ CFU/mL) by incubating the tubes for 16 h at 37 °C with shaking (150 rpm). The bacterial cultures were then used to inoculate either normal or modified BHI or DM broths at approximately 10^3^ CFU/mL, and the cultures were incubated at 3 or 4 °C without agitation or at 37 °C with shaking at 150 rpm. Growth of the strains was monitored by viable cell counts as previously described [41]. Four independent experiments and two replicates per experiment were conducted. Lag phases and growth rates were calculated using the DMFit program (version 2.1, Institute of Food Research, Reading, UK) [42]. The statistical significance of differences between the mutant strains and the wild-type strain in growth parameters were calculated using a Student’s t-test where *p*-values < 0.05 were considered significant. Global phenotypic analysis of the strains was performed using BIOLOG phenotypic microarrays (PMs) [43] according to the manufacturer’s protocol.

### 2.2. Motility Assays and Electron Microscopy

Swarming motility was determined by transferring 5 µL of an overnight culture of each strain onto the surface of trypticase soy broth (Difco Laboratories) solidified with 0.25% agar [44]. The plates were incubated at 3 and 37 °C for 6 weeks and 24 h, respectively.

The presence of flagella was examined using a Tecnai 12 transmission electron microscope (Philips Electron Optics, Eindhoven, The Netherlands) on BHI cultures grown to mid-logarithmic growth phase at 3 and 37 °C. Cells from 1 ml of each culture were fixed with 5% glutaraldehyde (Sigma-Aldrich, St. Louis, MO, USA), incubated at room temperature for 2 h and washed with 1 ml autoclaved water. The cells were then prepared on carbon-coated grids and negatively stained with 1% phosphotungstic acid hydrate (Sigma-Aldrich).

### 2.3. DNA Manipulation and Construction of the ΔsigB and ΔsigBL Strains

The *ΔsigB* and *ΔsigBL* mutants were constructed in this study based on previously described protocols [35,45]. Briefly, in-frame deleted copies of *sigB* and *sigL* genes were constructed using the splicing-by-overlap extension (SOE) PCR method [46] and primers listed in Table S1, and cloned into the temperature sensitive pKSV7 plasmid [47]. The pKSV7*ΔsigB* and pKSV7*ΔsigL* constructs were subsequently used in homologous-recombination-based replacement of the chromosomal copies of *sigB* and *sigL* genes, respectively, in the EGD-e and EGD-e*ΔsigL* strains using previously described experimental protocols [45]. Deletion mutants were confirmed through PCR amplification and subsequent sequencing of the targeted EGD-e DNA regions.

### 2.4. RNA Isolation and Gene-Expression Analysis

RNA isolation and gene-expression analysis using DNA microarrays and reverse transcription real-time PCR (RT-qPCR) were performed as previously described [36]. Primers used for gene expression by qRT-PCR are listed in Table S1. The differentially expressed genes in microarrays were defined as those with a ≥ 2.5-fold change in expression and a moderated *t*-test statistical significance of *p* ≤ 0.01. These genes were divided into functional categories based on annotations provided through the Comprehensive Microbial Resource of the J. Craig Venter Institute (Rockville, MD, USA) (http://cmr.jcvi.org). The microarray data described in this study were deposited in NCBI’s Gene Expression Omnibus (GEO accession number GSE32434) [48].

## 3. Results

### 3.1. Phenotypic Characterization of ΔsigB and ΔsigBL

We previously showed that the *ΔsigL* mutant impaired cold, salt, and organic acid stress growth phenotypes [36]. Growth phenotypes between *ΔsigB* and *ΔsigBL* mutants and their parental strain were compared (see Table 1). Both mutants were diminished during cold growth in DM but not in BHI compared to the EGD-e WT parent strain. In DM at 4 °C, the *ΔsigB* mutant displayed a prolonged lag phase. The *ΔsigBL* mutant displayed an even more prolonged lag phase as well as a slower growth rate compared to the parent strain under cold stress (Table 1 and Figure S1). The *ΔsigB* and *ΔsigBL* mutants were also altered in growth under different types of organic-acid stress. In the presence of lactic-acid stress, both *ΔsigB* and *ΔsigBL* grew with a lag phase delay compared to the parent strain (Table 1 and Figure S1). The prolonged lag phase in *ΔsigBL* was also accompanied by a slower growth rate compared to the parent strain under lactic-acid stress in BHI at 4 °C. Assessing growth under acetic-acid stress revealed that the *ΔsigB* mutant had a prolonged lag phase compared to the parent strain, whereas the *ΔsigBL* mutant completely failed to grow. Although the *ΔsigB* mutant had similar growth to the parent strain, the *ΔsigBL* mutant was unable to grow under citric-acid stress in BHI at 4 °C. Moreover, both mutants were characterized by longer lag phases and slower growth rates than the parent EGD-e WT strain when exposed to ethanol stress (5%) in DM at 37 °C (Table 1 and Figure S1).

On PMs, we previously showed diminished growth of the *ΔsigL* mutant on N-acetyl-D-Glucosamine as carbon sources, as well as under low pH (pH 5.0) and osmotic stress conditions [32]. The *ΔsigB* mutant displayed no phenotypic growth differences on carbon source utilization PMs (PM01 and PM02), whereas the *ΔsigBL* mutant had diminished growth compared to the parent strain on N-Acetyl-D-Glucosamine (PM01) and 3-0-b-D-Galactopyranosyl-D-Arabinose (PM02). Even though osmotic and acid stress tolerance are known to be *sigB*-dependent traits in *L. monocytogenes*, there were no phenotypic growth defects detected between the *ΔsigB* and WT strains under conditions of the osmotic or low pH stress sensitivity PMs (PM09–PM10) applied. The *ΔsigBL* mutant, on the other hand, displayed increased sensitivity compared to the parental strain under such osmotic and low pH stress conditions. Apart from that, both *ΔsigB* and *ΔsigBL* mutants displayed increased sensitivity to various chemical compounds when compared to the parental EGD-e strain on chemical sensitivity PMs (PM11–20). The *ΔsigB* strain exhibited increased sensitivity to seven chemical compounds (Table S2), whereas the *ΔsigBL* strains showed increased sensitivity to several compounds including those targeting cell-wall and protein synthesis, respiration, and DNA and RNA metabolism (Table S3). Notably, there were several additional chemical sensitivity phenotypes detected in the *∆sigBL* mutant that were undetected in *ΔsigB* or *ΔsigL* single deletion mutants (Table S4).

The swarming motilities of the *ΔsigB* and *ΔsigBL* mutants and the parent strain were determined on semisolid swarming agar and compared to those of the *ΔsigL* mutant. In contrast to the *sigL* null mutant, which was non-motile, both *sigB* and *sigBL* null mutants displayed similar zones of swarming motility to the parental EGD-e strain during incubation on soft agar surface at 3 °C. At 37 °C, the wild-type EGD-e strain and both mutant strains were non-motile. Further electron microscopic examination of these strains revealed that the parent strain and the *ΔsigB* and *ΔsigBL* mutants are flagellated, whereas the *ΔsigL* strain in contrast was non-flagellated in cold growth at 3 °C. Although flagellation patterns in *ΔsigB* and the parent EGD-e strain were similar, the amount of flagella per cell observed in the *ΔsigBL* strain was lower. At 37 °C, none of the strains formed flagella (Figure S2).

### 3.2. Identification of Genes de-Regulated in ΔsigB and ΔsigBL Mutants

**Figure 1:**
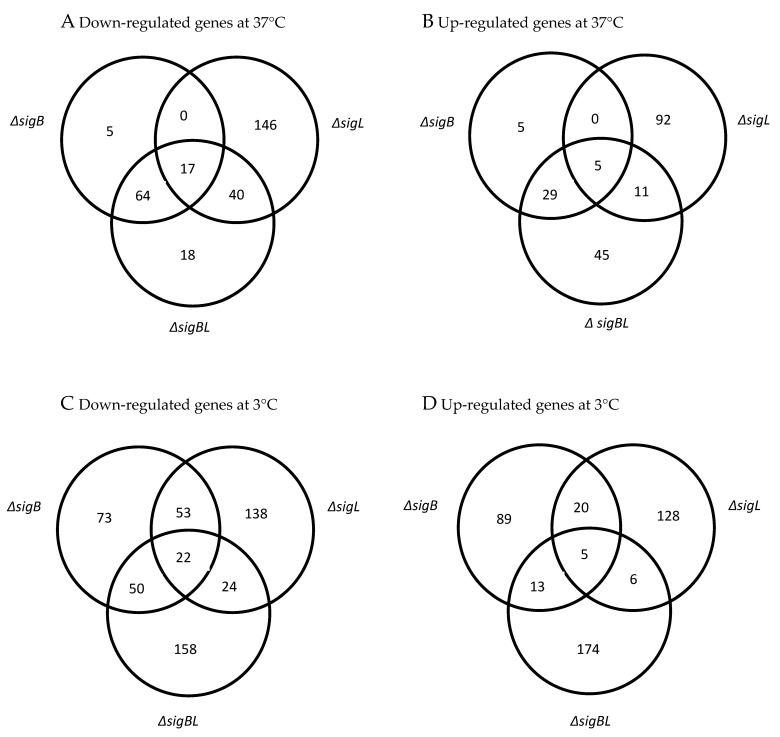
Overview of down- and up-regulated genes in *Listeria monocytogenes ΔsigB*, *ΔsigL* [36] and *ΔsigBL* compared to the wild-type EGD-e strain detected during exponential growth in BHI at 37°(**A**) and (**B**) and 3 °C (**C**) and (**D**). Genes showing 2.5-fold change (*p*-value ≤ 0.01) in transcript abundance were considered differentially expressed.

**Figure 2:**
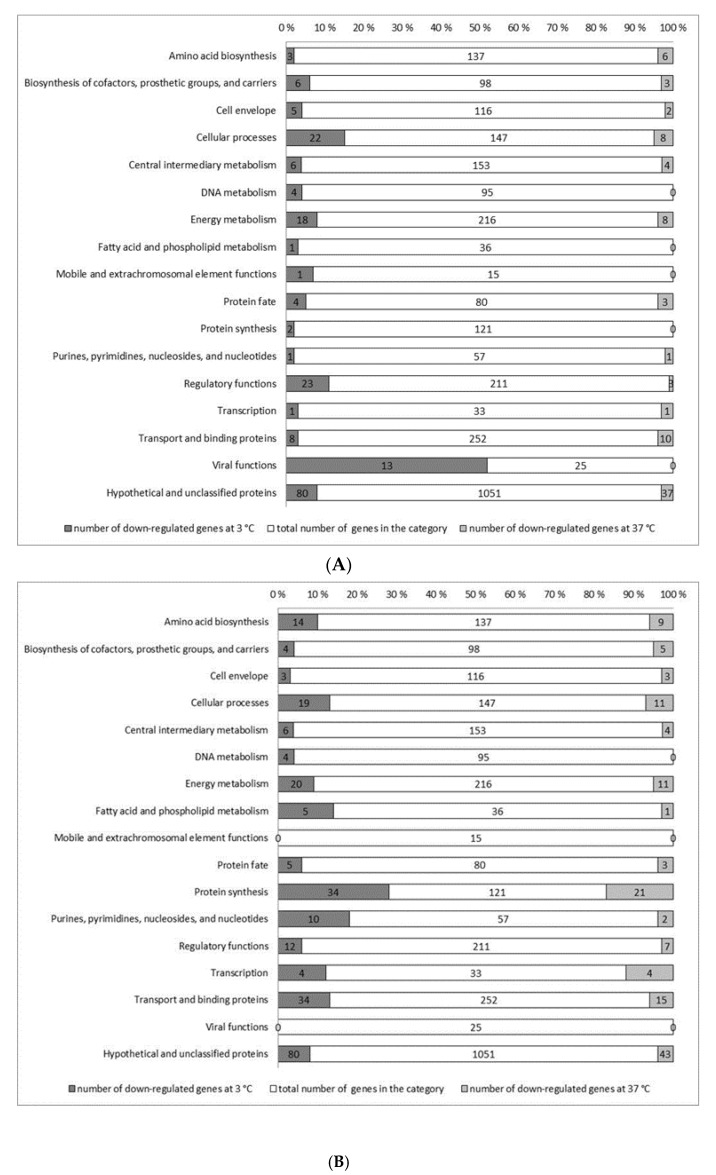
Distribution of significantly down-regulated genes in *Listeria monocytogenes ∆sigB* (**A**) and *∆sigBL* (**B**) during exponential growth in BHI at 3 and 37 °C in functional categories. The bar lengths represent the percentage of affected and unaffected genes assigned to each functional category. The genes were grouped into functional categories based on the annotations provided by the Comprehensive Microbial Resource of the J. Craig Venter Institute (CMR-JVCI) (http://cmr.jcvi.org).

Genome-wide microarrays were used to determine genes whose transcripts are de-regulated in *ΔsigB* and *ΔsigBL* mutants during exponential growth in BHI under cold (3 °C) and optimized (37 °C) temperature conditions. Based on an assigned *p*-value of ≤ 0.01 and 2.5-fold cutoff, 787 genes were considered differentially expressed in these two mutants compared to the parental strain (Figure 1). Of these differentially expressed genes, those deregulated in the *ΔsigB* mutant comprised 325 (127 up- and 198 down-regulated) at 3 °C and 125 (39 up- and 86 down-regulated) at 37 °C. Those deregulated in *ΔsigBL* compared to the parental EGD-e strain included 452 (198 up- and 254 down-regulated) at 3 °C, and 229 (90 up- and 139 down-regulated) at 37 °C. The transcriptome microarray data were validated on a selection of genes (*lmo0096*, *lmo0137*, *lmo0685,* and *lmo2625*) using quantitative real-time reverse-transcription PCR (RT-qPCR). The expression levels of this gene selection in the microarray data were in line with the results obtained with RT-qPCR (Table 2). The current study focuses on the set of those genes that were found down-regulated in *ΔsigB* and *ΔsigBL* mutant strains. This group comprises 284 (198 at 3 °C and 86 genes at 37 °C) genes identified in *ΔsigB* and 393 (254 at 3 °C and 139 at 37 °C) genes identified in *ΔsigBL*. Genes in this set with known functions were assigned biological functional categories showing that loss of σ^B^ and σ^L^ functions is associated with decreased transcription of genes that are involved in various cellular functions (Figure 2 and Tables S5–S8).

### 3.3. Genes Down-Regulated in ∆sigB During Exponential Growth in BHI at 3 °C

Altogether, 198 genes were down-regulated in the *∆sigB* mutant during exponential cold growth including 80 (40.4%) hypothetical and 118 (59.6%) functionally classified genes. The majority of these genes are involved in transcription regulation (12%), cellular processes (11%), energy metabolism (9%), and viral-associated functions (6%). The viral functions (52%), cellular processes (15%), regulatory functions (11%), and energy metabolism (8%) were categories with the largest percentage of down-regulated genes (Figure 2).

Genes showing the largest down-regulation during exponential cold growth in the *∆sigB* mutant were *lmo2158* and *lmo0433*, which code for a conserved domain protein and internalin A, respectively. Meanwhile, there were 22 EGD-e polycistronic operons that had all their genes down-regulated in *∆sigB* during exponential cold growth [49]. These include: operons 123 and 124 comprising *lmo0781–lmo0784* genes, which encode a mannose-specific phosphotransferase system (PTS), and operons 168 and 169 comprising *lmo1041–lmo1048* genes, which are predicted to code for molybdenum transport and molybdenum and molybdopterin biosynthesis proteins. Other genes also found among those down-regulated in this mutant include *lmo1172–lmo1173*, and *lmo2582–lmo2583*, which code for bacterial two-component signaling system proteins. Furthermore, operon 415, consisting of 15 bacteriophage A118-associated protein encoding genes *lmo2278–lmo2301*, had 13 genes that were down-regulated during cold growth in the *sigB* null mutant.

### 3.4. Genes Down-Regulated in ∆sigB During Exponential Growth in BHI at 37 °C

There were 86 genes down-regulated in *∆sigB* relative to the parental strain during exponential growth at 37 °C. Of these genes, 50 (58.1%) encode functionally classified proteins, whereas 36 (41.9%) encode hypothetical proteins (Table S6). Genes from the categories of transport and binding (12%), cellular processes (9%), energy metabolism (9%) and amino acid biosynthesis (7%) predominated. The biological function categories of cellular processes (5%), amino acid biosynthesis (4%), energy metabolism (4%), protein fate (4%), and transport and binding proteins (4%) had relatively the largest percentages of genes in this set (Figure 2).

In 10 of the EGD-e polycistronic operons, all genes were repressed in the *∆sigB* mutant including operons 016, consisting of genes *lmo0096–lmo0098*, 123, and 124; consisting of genes *lmo0781–lmo0784* that code fructose and mannose specific PTS systems; and operon 022 consists of genes *lmo0135–lmo0137,* which encodes ABC transport-system proteins. Operon 461 that comprises genes *lmo2570–lmo2573,* which is predicted to encode proteins that are part of the nicotinate and nicotinamide metabolism pathway, was also down-regulated. Genes *lmo0263* and *lmo2158*, which code for internalin H and a conserved domain protein, respectively, were the most down-regulated in this set.

There were 29 genes subjected to positive σ^B^ transcriptional regulation during EGD-e exponential growth in BHI at 3 and 37 °C that were unveiled in the current study. These genes include those coding for PTS components, internalin H, universal stress, and pyruvate metabolism proteins (Table 3).

### 3.5. Genes Down−Regulated in ∆sigBL during Exponential Growth in BHI at 3 °C

There were 254 down−regulated genes detected in *∆sigBL* during exponential cold growth (Table S7). Eighty (31.5%) such genes encode hypothetical proteins, whereas 174 (68.5%) encode proteins with confirmed or predicted functions, the majority of which belong to transport and binding proteins (13%), protein synthesis (13%), energy metabolism (8%), and cellular processes (7%) categories. Protein synthesis (28%), purines, pyrimidines, nucleosides and nucleotides (18%), fatty acid and phospholipid metabolism (14%), cellular processes (13%), and transport and binding proteins (13%) were the categories with the largest percentages of down−regulated genes found in this group (Figure 2).

In 20 EGD−e polycistronic operons, all genes were down−regulated in the *∆sigBL* mutant during exponential cold growth. These included operons 022 comprising *lmo0135–lmo0137*, 305 comprising *lmo1738–lmo1740*, and 326 consisting of genes *lmo1847–lmo1849*, all encoding ABC transport−system proteins. Operons 068 comprising *lmo0398–lmo0402* genes, and 123 1nd 124 consisting of genes *lmo0781–0784* that encode fructose− and mannose−specific PTS system components were also down−regulated. Genes *lmo1406–lmo1407* in the down−regulated operon 229 encode for the pyruvate formate−lyase (PflB) and pyruvate formate−lyase activating enzyme (PflC), which are part of the pyruvate metabolism pathway. Genes *lmo1538–lmo1539* in down−regulated operon 258 encode glycerol kinase and glycerol uptake facilitator protein needed in glycerolipid metabolism. The *lmo1983–lmo1991* genes in down−regulated operon 360 encode *ilvD, ilvB, ilvH, ilvC, leuA, leuB, leuC, leuD,* and *ilvA,* which are all part of the pyruvate gene−family. Genes *lmo2201–lmo2202* in down−regulated operon 399 encode 3−oxoacyl synthase II and 3−oxoacyl synthase III, which are needed in fatty acid metabolism and biosynthesis. The down−regulated operons 042 (*lmo0248–lmo0248*), 043 (*lmo0250–lmo0251*), and 315 (*lmo1796–lmo1797*) consist of ribosomal protein encoding genes. Overall, 27 of the 57 known ribosomal genes in *L. monocytogenes* EGD−e genome were down−regulated in *∆sigBL*.

The genes showing the largest down−regulation in *∆sigBL* compared to the wild−type strain during exponential growth in BHI at 3 °C were *lmo1634*, which encodes aldehyde−alcohol dehydrogenase and *lmo1847*, which encodes endocarditis specific antigen.

Genes that were down−regulated during exponential cold growth in *∆sigBL,* but not in *∆sigB* or *∆sigL* [32] single deletion mutants, are shown in Table S9.

### 3.6. Genes Down−Regulated in ∆sigBL During Exponential Growth in BHI at 37 °C

Altogether 139 genes were down−regulated in *∆sigBL* during exponential growth in BHI at 37 °C (Table S8). Of these genes, 43 (30.9%) encode hypothetical proteins and 96 (69.1%) code for proteins with confirmed or predicted functional roles. Most down−regulated genes belonged to protein synthesis (15%), transport and binding proteins (11%), energy metabolism (8%), and cellular processes (8%) functional categories. The largest percentages of down−regulated genes per functional category were detected in protein synthesis (17%), transcription (12%), cellular processes (7%), amino acid biosynthesis (6%), and transport and binding proteins (6%) (Figure 2).

Down−regulated genes included 12 polycistronic operons in which all genes were down−regulated. Besides previously described operons 016, 123, 124, and 022 comprising genes coding PTS and ABC transport−system proteins, other operons among these were 077 consisting of genes *lmo0433–lmo0434*, internalin A encoding gene *inlA*, and internalin B encoding *inlB*. Operon 461, which also had all genes down−regulated, comprises *lmo2570–lmo2573* genes that are predicted to play a role in nicotinate and nicotinamide metabolism. Similar to cold growth at 3 °C, 22 of the 57 EGD−e ribosomal genes were down−regulated in *∆sigBL* during exponential growth at 37 °C. Genes *lmo0098* and *lmo0096* encoding PTS−system components were the most down−regulated genes detected in *∆sigBL* during exponential growth at 37 °C.

Sixty−three genes were down−regulated during exponential growth of *L.monocytogenes ΔsigBL* in BHI at both 3 and 37°C. These genes are presented in Table 4. Genes down−regulated in *L.monocytogenes ∆sigBL,* but not in *∆sigB* or *∆sigL* [32], during exponential growth in BHI at 37 °C are shown in Table S10.

## 4. Discussion

Alternative sigma factors are used as transcriptional regulators of various gene−expression responses in bacteria. They are used to regulate various cellular processes needed for survival and growth of bacteria under favorable and non−favorable conditions [50,51,52,53,54]. Since σ^B^ and σ^L^ are important regulators of stress adaptation responses in *L. monocytogenes,* we have further investigated their functional contributions to growth under stress conditions and gene expression regulation in *L. monocytogenes* EGD−e. Phenotypic evaluation of the *∆sigB* and *∆sigBL* mutants revealed impaired growth in the presence of organic acids and ethanol, as well as altered flagellation at low growth temperatures. In addition, significant differences in growth of the *sigB* and *sigBL* null mutant strains compared to the parent strain on different carbon sources and in the presence of various chemical compounds were revealed. A transcriptome comparison between the *∆sigB* and *∆sigBL* mutants and the parental EGD−e strain during exponential cold (3 °C) and optimal (37 °C) temperature growth established that the expression of many of genes are de−regulated in these mutants.

### 4.1. Identification of σ^B^ Positively Regulated Genes during Exponential Growth in BHI at 3 and 37 °C 

The current study identified 284 genes that are positively regulated in a σ^B^−dependent manner during exponential growth of *L. monocytogenes* EGD−e—198 at 3 °C and 86 at 37 °C. These genes were compared to previously identified σ^B^ positively regulated genes. Kazmierczak et al. [14] identified 55 and Raengpradub et al. [17] 168 σ^B^ positively regulated genes in *L. monocytogenes* 10403S cells grown to stationary phase or under salt stress. Thirty three (17 at 3 °C and 16 at 37 °C) genes detected here overlapped with those reported by Kazmierczak et al. [16], whereas 103 (45 at 3 °C and 58 at 37 °C) genes were among those identified by Raengpradub et al. [19]. Ninety−one genes (40 at 3 °C and 51 at 37 °C) detected here were among the 105 genes of the σ^B^ regulon described by Hain et al. [50] in EGD−e grown in BHI at 37 °C. Oliver et al. [27] described a σ^B^ core regulon that consists of at least 63 genes using lineage I, II, IIIA, and IIIB strains. Out of these, there were 27 and 38 genes that were also detected in our study at 3 and 37 °C, respectively. All 15 σ^B^ positively regulated genes, recently identified by combining microarray, proteomics, and RNA−sequencing [30], were also detected in our study.

The mannose−specific phosphotransferase system (PTS) operon (*lmo0781–lmo0784*) that was shown to be positively regulated by σ^B^ at both growth temperatures in this study was among σ^B^−dependent genes described by Kazmierczak et al. [16], Raengpradub et al. [19], and Hain et al. [55]. The *lmo2570–lmo2573* genes belonging to the nicotinate and nicotinamide metabolism pathway detected in our study at 37 °C were previously also detected among σ^B^–dependent genes reported by Kazmierczak et al. [16] and Hain et al. [55]. Among other genes, our study found that genes from the fructose− and mannose−specific PTS−encoding operon 016 (*lmo0096–lmo0098*) are also positively regulated through σ^B^ during cold growth at 3 °C, which had not been previously reported from other studies.

Operons 168 and 169, which encode molybdenum transport and molybdenum and molybdopterin biosynthesis proteins, were shown by the current study to be positively regulated through σ^B^ during *L. monocytogenes* cold growth. Transcription from the *moa* locus that encodes enzymes required for molybdopterin biosynthesis in *Escherichia coli* is enhanced under anaerobic conditions [56]. Our transcriptome findings suggest that molybdopterin genes in *L. monocytogenes* may be involved in cold−growth functions, but additional future experiments are needed to examine such a role.

σ^B^−dependent positive control of operon 415 comprising bacteriophage A118 associated protein−encoding genes was detected during cold growth at 3 °C. Similar regulation of these phage genes in a σ^L^−dependent manner was detected in our previous study with the *sigL* null mutant [36]. Overall, the comparison of our data to previously described σ^B^ regulons is limited by differences in the cut−off criteria used to define σ^B−^regulated genes as well as the use of different strains and growth conditions.

### 4.2. Stress Response Phenotypic Traits Associated with a ∆sigB Mutation in L. Monocytogenes EGD−e

A comparison of growth phenotypes between parental and *∆sigB* EGD−e strain revealed that this mutant has compromised growth under lactic−acid, acetic−acid, and ethanol stresses. Our observations here are in agreement with previous studies describing reduced acid−stress tolerance in *L. monocytogenes sigB* null mutants [15,17,57,58]. Ferreira et al. [57] previously showed that σ^B^ is not essential for *L. monocytogenes* viability under lethal ethanol (16.5%) stress. Our study shows, however, that σ^B^ is essential for optimal growth of this bacterium in the presence of sublethal ethanol (5%) stress under restricted nutrient conditions in minimal defined medium.

Although osmotic and acid stress resistance are well established as *sigB*−dependent traits in *L. monocytogenes*, we detected no phenotypic defects between *∆sigB* and the parental strain on the osmotic or low−pH stress sensitivity arrays in phenotypic microarray analysis. Reasons for these observations are not yet known, but it is possible that there are differences between PM assay conditions and those previously used in other studies, and the conditions in the PM array might have masked the osmotic and acid stress defects in the *∆sigB* mutant in the present study.

No differences were detected in flagellation and swarming ability between the *∆sigB* mutant and its parent strain during cold growth at 3 °C. Consistent with this, transcriptomic analysis also showed that the *∆sigB* mutation does not substantially affect the expression of flagella− and motility−associated genes during cold growth. Hu et al. [59] previously reported similar observations concerning the swarming ability of *L. monocytogenes* strain 10403S and its *∆sigB* mutant grown at room temperature.

### 4.3. Impact of the ∆sigBL Mutation on the L. Monocytogenes EGD−e Transcriptome

In comparison to the parental strain, at 3 °C, 254 genes were down−regulated and 198 genes were up−regulated in the exponentially growing *ΔsigBL* strain cultivated in BHI. Comparatively, at 37 °C, this mutant had 139 genes down−regulated and 90 genes up−regulated compared to the parent strain.

Of the 254 genes down−regulated in *ΔsigBL* at 3 °C, 158 (62%) were not detected under similar conditions in a *∆sigB* mutant during this study or the *∆sigL* mutant we previously analyzed [36]. On the other hand, 18 (13%) of 139 down−regulated genes were exclusively detected in *ΔsigBL* at 37 °C. Our observations thus indicate that while cold growth stress expands the specialized expression profile of *ΔsigBL,* the expression profile of this mutant resembles more those of the *ΔsigB* and *ΔsigL* mutants during growth at optimal temperature.

Genes *lmo1152–lmo1167* encoded by the putative propanediol utilization (*pdu*) operon were amongst genes exclusively down−regulated in *ΔsigBL* during exponential cold growth. Apart from that, the predicted *ilv−leu* operon 360 coding for IlvD, IlvB, IlvH, IlvC, LeuA, LeuB, LeuC, LeuD, and IlvA*,* involved in the synthesis of branched−chain amino acids (valine, isoleucine, and leucine), was also exclusively down−regulated in the *ΔsigBL* background but not in *ΔsigB* or *ΔsigL* single deletion mutants at 3 °C. Garmyn et al. [60] previously compared the *L. monocytogenes* EGD−e transcriptomes at 25 and 37 °C and found that genes in both *pdu* and *ilv−leu* operons were up−regulated at 25 °C compared to 37 °C. Recent studies have suggested that the Pdu cluster may support the growth of this pathogen in specific stress conditions along the food chain [61,62]. Tojo et al. [63] reported repression expression from the *ilv−leu* operon during growth under nitrogen−limited conditions in *Bacillus subtilis*. Individual or co−regulatory effects of σ^B^ and σ^L^ in transcription of *pdu* and *ilv−leu* operons in *L. monocytogenes* have not been previously investigated.

Nine of the 12 genes in operon 310 comprising purine ribonucleotide biosynthesis genes *purD, purG, purN, purM, purF, purQ, purl, lmo1771, purC, purB, purK,* and *purE* were repressed in *∆sigBL* at 3 °C but not at 37 °C or in *ΔsigB* or *ΔsigL*. To our knowledge, the link between σ^B^ and σ^L^ in temperature−dependent regulation of the *pur* operon in *L. monocytogenes* has not been described previously.

Other genes we found to be down−regulated at both 3 and 37 °C in the *sigBL* null mutant background included *inlH* and *inlA*, 17 ribosomal genes, operons 123− and 124−encoding proteins belonging in the PTS system, and fructose and mannose metabolic pathway and operon 022 encoding protein parts of the ABC transport system. All these genes are assumed to be down−regulated in a temperature−independent manner in the *sigBL* null mutant.

Operon 068, encoding PTS−system related and fructose− and mannose−specific genes, was down−regulated in *∆sigBL* at 3 °C but not at 37 °C in this study, and it was also recently reported to be co−regulated through multiple transcription factors, including σ^B^ and σ^L^ [28]. Operon 305, which encodes predicted ABC transport−system proteins, showed a similar temperature−dependent expression profile in both *∆sigL* and *∆sigBL* mutant strains.

Several ribosomal genes were down−regulated in the *∆sigBL* mutant at both growth temperatures while in the *∆sigL* mutant these genes were down−regulated only at 37 °C. Several transcripts of bacteriophage A118 associated protein encoding genes were down−regulated in the *sigB* null mutant at 3 °C and in the *sigL* null mutant at both growth temperatures. Repression of these genes was lost at both 3 and 37 °C in the *sigBL* null mutant background.

The number of studies performed to date using *L. monocytogenes ∆sigBL* mutant strains remains low, and as such, knowledge on the gene expression profile in this alternative sigma factor double−mutant strain is also limited. Palmer et al. [64] studied the contributions of σ^B^ and σ^L^ to *L. monocytogenes* response to antimicrobial substance—nisin—and to transcription of putative bacteriocin immunity gene *lmo2570*. The gene *lmo2570* was found to be regulated by σ^B^ but not by σ^L^. In line with these observations, our studies found that the gene *lmo2570* was down−regulated in *∆sigB* and *∆sigBL* mutants at 37 °C but not in *∆sigL* mutant.

### 4.4. Stress Response Phenotypic Traits Associated with σ^B^and σ^L^ Inactivation in L. Monocytogenes EGD−e 

Growth phenotypes in *∆sigBL* were compared to the parental strain as well as *∆sigB* and *∆sigL* strains under different environmental stress conditions. Growth of the *∆sigBL* mutant was drastically compromised in the presence of cold, lactic−acid, acetic−acid, citric−acid, and ethanol stress conditions relative to the parent strain. In our previous study [36], the *ΔsigL* strain showed similar growth phenotype defects exposed to cold, lactic−acid, acetic−acid, citric−acid, and ethanol stresses. Overall, the growth of the *ΔsigBL* mutant in acid, ethanol, and cold stresses better resembled the phenotype of the *ΔsigL* than the phenotype of *ΔsigB.* In a previous study [64], the *∆sigB∆sigL* mutant of *L. monocytogenes* 10403S showed a significantly slower growth rate after exposure to nisin compared to the wild−type strain and *∆sigB* and *∆sigL* mutants. Apart from this, to our knowledge, no other studies on environmental stress growth with *L. monocytogenes ΔsigBL* mutants have yet been reported.

Phenotypic microarray analysis revealed 24 different compounds that impaired growth of the *∆sigBL* strain compared with the wild−type, and that did not affect the growth of the *∆sigB* or *∆sigL* [36]. Most of these chemical compounds were antibiotics targeting cell wall, membrane, DNA, or protein synthesis (Table S4). Consistent with some of these phenotypes, there was a down−regulation in expression of transcripts encoding cell−membrane and protein−synthesis−associated proteins detected in the *∆sigBL* at 37 °C.

The transcriptomic analysis also showed that deletion in both *sigB* and *sigL* affected the expression of four flagella−associated genes in cells grown at 3 °C. The flagellin coding gene *lmo0690* showed the largest down−regulation, 12.3−fold decrease compared to the parent strain, while the *flgC, fliE,* and *flgB* all showed modest down−regulation with fold decreases ranging between 2.0 and 2.6.

Although the zone of swarming displayed by the *∆sigBL* strain did not differ significantly from that of the parent strain, electron microscopy showed that the number of flagella per cells in this mutant was reduced compared to the parent strain. Evidently, the swarming ability of the *∆sigBL* strain is unaffected by discreet down−regulation of these four genes, but flagella formation is nevertheless reduced.

In the *ΔsigL* mutant strain [36], neither flagellation nor swarming motility were detected. Interestingly, the double deletion of *sigB* and *sigL* restores both flagellation and motility in the *ΔsigBL* strain. These phenotypic observations are supported by the transcriptomic data since the deletion in both *sigB* and *sigL* affected the expression of only four flagella−associated genes in cells grown at 3 °C compared to the parent strain, whereas in the *ΔsigL* strain, 19 genes related to flagella biosynthesis, chemotaxis, and motility processes were down−regulated. Chaturongakul et al. [28] also discussed the coordinated and differential regulation of *L. monocytogenes* motility and chemotaxis genes under different growth conditions by multiple regulators, including σ^B^ and σ^L^. A *ΔsigB* mutation increases flagella expression and motility in part due to decreased *mogR* expression, which relieves the repression of flagella expression. The *mogR* repressor is controlled by two promoters, including the P1 promoter, which is σ^B^−dependent [44].

Deletion in both *sigB* and *sigL* increases the sensitivity of the *L. monocytogenes* EGD−e towards different stress conditions and chemical compounds, and promotes phenotypic characteristics absent in either the *sigB* or *sigL* null mutant strain. The interactive effect detected after the loss of both *sigB* and *sigL* may indicate that at least some genes needed for a sufficient stress response of *L. monocytogenes* EGD−e are co−regulated either directly or indirectly by these alternative sigma factors. As such, it appears the stress response mechanisms that are positively regulated through σ^B^ and σ^L^ co−regulation might be more relevant in *L. monocytogenes* stress protection than what has been known to date.

The present study provides an expanded insight into σ^B^ and σ^L^ phenotypic roles and functional interactions in *L. monocytogenes*. Besides previously known σ^B^− and σ^L^−dependent genes, the transcriptomes defined in *ΔsigB* and *ΔsigBL* mutants reveal several new genes that are positively regulated by σ^B^ alone, as well as those co−regulated through σ^B^− and σ^L^−dependent mechanisms during *L. monocytogenes* growth under optimal and cold−stress temperature conditions. Future studies in this area should include validation of the σ^B^ and σ^L^ co−regulation of such genes as well as their functional characterization through deletion mutagenesis and phenotypic analysis of these mutants. Moreover, the mechanistic aspects of how σ^B^− and σ^L^−dependent co−regulation of such target genes is achieved will be essential in providing insights into the σ^B^ and σ^L^ regulatory networks in *L. monocytogenes.*

## Figures and Tables

**Table 1:** Growth parameters determined for *Listeria monocytogenes ΔsigB, ΔsigBL*, and parental EGD-e strain under different conditions in brain heart infusion broth (BHI) and defined medium (DM) at 4 and 37 °C.

Growth Condition	Growth Parameter	WT	*ΔsigB*	*ΔsigBL*	Statistical Significance
4 °C BHI (pH 7.4)	LPD (wk) GR (Log_10_(CFU/mL)/wk)	0.00 0.89 ± 0.03	0.00 0.88 ± 0.04	0.00 0.85 ± 0.04	NS ^a^ NS
BHI plus 2.5% lactic acid (pH 6.0)	LPD (wk) GR (Log_10_(CFU/mL)/wk)	3.84 ± 0.10 1.01 ± 0.01	***4.51 ± 0.15*** ***1.10 ± 0.02***	***5.48 ± 0.67*** ***0.32 ± 0.01***	S ^b,c^ S
BHI plus 0.35% acetic acid (pH 5.5)	LPD (wk) GR (Log_10_(CFU/mL)/wk)	2.10 ± 1.32 0.13 ± 0.01	***7.33 ± 0.47*** ***0.23 ± 0.03***	***NG*** ***NG***	S S
BHI plus 0.35% citric acid (pH 5.5)	LPD (wk) GR (Log_10_(CFU/mL)/wk)	3.98 ± 0.96 1.11 ± 0.15	***2.82 ± 0.10*** 1.21 ± 0.11	***NG*** ***NG***	S S
DM	LPD (wk) GR (Log_10_(CFU/mL)/wk)	2.44 ± 0.18 1.27 ± 0.07	***2.87 ± 0.05*** ***1.63 ± 0.11***	***4.60 ± 0.27*** ***0.49 ± 0.17***	S S
37 °C DM	LPD (wk) GR (Log_10_(CFU/mL)/wk)	0.00 0.14 ± 0.01	0.00 0.12 ± 0.01	0.00 0.11 ± 0.03	NS NS
DM plus 5% Ethanol	LPD (wk) GR (Log_10_(CFU/mL)/wk)	5.87 ± 2.51 0.12 ± 0.02	***17.79 ± 1.78*** **0.04 ± 0.05**	***14.27 ± 1.58*** **0.03 ± 0.02**	S S

The lag phases (LPD) and growth rates (GR) of the strains were estimated using the Dmfit program (version 2.1), which is based on the Baranyi model. NG—no exponential growth detected. ^a^ NS—No statistically significant differences between ***Δ****sigB* and ***Δ****sigBL* compared to the EGD-e WT strain. ^b^ S—Statistically significant differences (*p* < 0.05) between ***Δ****sigB* and ***Δ****sigBL* compared to the EGD-e WT strain are indicated in bold. ^c^ S—Statistically significant differences (*p* < 0.05) between ***Δ****sigB* and ***Δ****sigBL* strains are indicated in bold and italics. The statistical significance information is based on one-way ANOVA comparison of the mutants to the parental WT strain.

**Table 2:** Validation of selected DNA-microarray results with real-time RT-qPCR.

Gene	37 °C				3 °C			
	*ΔsigB*		*ΔsigBL*		*ΔsigB*		*ΔsigBL*	
	Microarray	RT-qPCR	Microarray	RT-qPCR	Microarray	RT-qPCR	Microarray	RT-qPCR
*lmo0096*	▼	▼	▼	▼	▲	▲	NS	NS
*lmo0137*	▼	▼	▼	▼	NS	NS	▼	▼
*lmo0685*	NS ^a^	NS	NS	NS	NS	NS	▼	▼
*lmo2625*	▼	▼	▼	▼	NS	NS	▼	▼

Relative gene expression levels in deletion mutant strains *ΔsigB, ΔsigL*, and *ΔsigBL* compared with the EGD-e parent strain. ▼ indicates down-regulation and ▲ up-regulation of the gene in the mutant strain compared with the wild-type. ^a^ No statistical difference in the gene expression levels in the mutant strains compared with the parent strain.

**Table 3:** Genes that were down-regulated during exponential growth of *Listeria monocytogenes ΔsigB* in BHI at 3 and 37 °C ^a^.

Gene	Functional Category and Protein ^b^	Differential Expression (∆*sigB* vs. WT) Fold Change at 3 °C	Differential Expression (∆*sigB* vs. WT) Fold Change at 37 °C
	**Amino acid biosynthesis**		
*lmo0781*	PTS system component	−1.5	−2.3
	**Cell envelope**		
*lmo1079*	membrane protein, putative	−1.4	−2.1
	**Cellular processes**		
*lmo0263*	internalin H	−3	−4.1
*lmo0515*	universal stress protein family	−2.9	−2.5
*lmo0669*	general stress protein 39	−1.8	−3.5
*lmo1694*	cell division inhibitor	−1.5	−2.4
*lmo2230*	arsenate reductase, putative	−4	−3.9
*lmo2673*	universal stress protein family	−2.4	−2.8
	**Central intermediary metabolism**		
*lmo0134*	acetyltransferase, GNAT family	−2.4	−1.4
*lmo2434*	glutamate decarboxylase	−3.2	−3.1
	**Energy metabolism**		
*lmo0722*	pyruvate oxidase	−2.3	−2.4
*lmo0913*	succinate−semialdehyde dehydrogenase	−2.5	−1.6
*lmo2674*	ribose 5−phosphate isomerase B	−1.8	−1.6
	**Protein fate**		
*lmo1407*	pyruvate formate−lyase activating enzyme	−1.4	−1.5
*lmo2157*	secretory protein (sepA)	−1.4	−2.2
	**Regulatory functions**		
*lmo2085*	Gram positive anchor domain protein	−3.3	−3
	**Transcription**		
*lmo0895*	RNA polymerase sigma factor B	−5.8	−5.3
	**Transport and binding proteins**		
*lmo0169*	transporter, putative	−2.1	−2.4
*lmo0782*	PTS system component	−3	−3.4
*lmo0783*	PTS system component	−3.3	−2.8
*lmo0784*	PTS system component	−3.7	−3.2
	**Hypothetical and unclassified proteins**		
*lmo0265*	peptidase, M20/M25/M40 family	−2.2	−2.4
*lmo0602*	protease synthase and sporulation negative regulatory protein pai 1, putative	−1.3	−2.8
*lmo0937*	hypothetical protein	−3.2	−2.8
*lmo1241*	conserved hypothetical protein	−2.5	−3.9
*lmo2067*	choloylglycine hydrolase	−3.1	−3.5
*lmo2158*	conserved domain protein	−6.2	−3.9
*lmo2213*	conserved hypothetical protein	−1.6	−2.6
*lmo2748*	conserved hypothetical protein	−1.9	−3

^a^ The log_2_−transformed mean fold change values were calculated from three biological replicates. Genes listed are those displaying ≥ 2.5 fold (equivalent to ± 1.3 log_2_) change in transcript abundance between the *ΔsigB* and its parental EGD−e strain with a moderated *t*−test statistical significance of *p*−value ≤ 0.01. ^b^ Gene functional categories are based on the annotations provided by the Comprehensive Microbial Resource of the J. Craig Venter Institute (CMR-JCVI) (http://cmr.jcvi.org).

**Table 4:** Genes that were down−regulated during exponential growth of *Listeria monocytogenes ΔsigBL* in BHI at 3 and 37 °C ^a^.

Gene	Functional Category and Protein ^b^	Differential Expression (∆*sigBL* vs. WT) Fold Change at 3 °C	Differential Expression (∆*sigBL* vs. WT) Fold Change at 37 °C
	**Biosynthesis of cofactors, prosthetic groups, and carriers**		
*lmo2571*	pyrazinamidase/nicotinamidase, putative	−1.4	−3.4
	**Cellular processes**		
*lmo0263*	internalin H	−2.7	−4.1
*lmo0433*	internalin A	−3.8	−1.3
*lmo0515*	universal stress protein family	−3.5	−2.2
*lmo0669*	general stress protein 39	−1.7	−3.6
*lmo1694*	cell division inhibitor	−1.9	−3
*lmo2230*	arsenate reductase, putative	−3.1	−4.2
*lmo2673*	universal stress protein family	−1.4	−3.3
	**Central intermediary metabolism**		
*lmo0134*	acetyltransferase, GNAT family	−2.5	−3
*lmo2434*	glutamate decarboxylase	−3.1	−3.4
	**Energy metabolism**		
*lmo0210*	L−lactate dehydrogenase	−3.2	−1.3
*lmo0913*	succinate−semialdehyde dehydrogenase	−2.1	−1.6
*lmo1634*	aldehyde−alcohol dehydrogenase	−6.1	−1.7
*lmo2674*	ribose 5−phosphate isomerase B	−2	−1.6
	**Protein fate**		
*lmo1407*	pyruvate formate−lyase activating enzyme	−3.3	−1.5
	**Protein synthesis**		
*lmo0250*	ribosomal protein L10	−2.2	−1.3
*lmo0251*	ribosomal protein L7/L12	−2.6	−1.4
*lmo1783*	ribosomal protein L20	−1.5	−1.3
*lmo2605*	ribosomal protein L17	−1.4	−1.6
*lmo2613*	ribosomal protein L15	−1.3	−1.4
*lmo2614*	ribosomal protein L30	−1.5	−2
*lmo2615*	ribosomal protein S5	−1.8	−2
*lmo2616*	ribosomal protein L18	−1.6	−1.9
*lmo2617*	ribosomal protein L6	−1.7	−1.7
*lmo2618*	ribosomal protein S8	−1.8	−1.7
*lmo2619*	ribosomal protein S14p/S29e	−1.6	−1.6
*lmo2620*	ribosomal protein L5	−1.9	−1.7
*lmo2621*	ribosomal protein L24	−1.7	−1.5
*lmo2622*	ribosomal protein L14	−1.5	−1.6
*lmo2624*	ribosomal protein L29	−1.9	−1.5
*lmo2625*	ribosomal protein L16	−1.8	−1.5
*lmo2626*	ribosomal protein S3	−1.6	−1.5
	**Purines, pyrimidines, nucleosides, and nucleotides**		
*lmo2611*	adenylate kinase	−1.5	−1.9
	**Regulatory functions**		
*lmo1956*	transcriptional regulator, Fur family	−1.5	−1.4
*lmo2085*	Gram positive anchor domain protein	−3.2	−3.6
	**Transcription**		
*lmo0895*	RNA polymerase sigma factor B	−2.3	−5.3
*lmo2461*	Sigma−54 factors family	−2.7	−3.7
	**Transport and binding proteins**		
*lmo0135*	oligopeptide−binding protein appa precursor, putative	−2.2	−2.8
*lmo0136*	peptide ABC transporter, permease protein	−1.8	−2.4
*lmo0137*	oligopeptide transport permease protein appc	−1.6	−2.6
*lmo0169*	transporter, putative	−1.3	−2.7
*lmo0781*	PTS system component	−2	−2.3
*lmo0782*	PTS system component	−2.2	−3.9
*lmo0783*	PTS system component	−2.1	−2.9
*lmo0784*	PTS system component	−2.1	−3.8
	**Hypothetical and unclassified proteins**		
*lmo0265*	peptidase, M20/M25/M40 family		
*lmo0355*	succinate dehydrogenase/fumarate reductase, flavoprotein	−2.2	−2.4
*lmo0602*	subunit	−1.9	−1.9
*lmo2067*	protease synthase and sporulation negative regulatory protein pai 1, putative	−2.1	−3
*lmo2158*	choloylglycine hydrolase		
*lmo0170*	conserved domain protein	−3	−3.6
*lmo0596*	conserved hypothetical protein	−4.7	−3.8
*lmo0911*	conserved hypothetical protein	−1.7	−1.5
*lmo0995*	conserved hypothetical protein	−1.6	−3.9
*lmo1241*	conserved hypothetical protein	−2	−1.6
*lmo1776*	conserved hypothetical protein	−2.3	−1.9
*lmo2213*	conserved hypothetical protein	−1.4	−4.2
*lmo2572*	conserved hypothetical protein	−2.8	−1.4
*lmo2748*	conserved hypothetical protein	−1.5	−2.8
*lmo0654*	conserved hypothetical protein	−1.4	−3.5
*lmo0937*	hypothetical protein	−2.5	−3.1
*lmo0994*	hypothetical protein	−1.3	−1.6
*lmo2454*	hypothetical protein	−3.3	−2.6

^a^ The log_2_−transformed mean fold change values were calculated from three biological replicates. Genes listed are those displaying ≥ 2.5 fold (equivalent to ± 1.3 log_2_) change in transcript abundance between the *ΔsigBL* and its parental EGD−e strain with a moderated *t*−test statistical significance of *p*−value ≤ 0.01. ^b^ Functional category—genes are functionally categorized based on the annotations provided by the Comprehensive Microbial Resource of the J. Craig Venter Institute (CMR−JCVI) (http://cmr.jcvi.org).

## Supplementary Materials

The following are available online at https://www.mdpi.com/2076-2607/8/11/1644/s1, Figure S1: Growth of Listeria monocytogenes EGD−e wild−type strain and *∆sigB* and *∆sigBL* mutant strains in BHI broth at 4 °C, in DM at 4 °C, in BHI supplemented with lactic acid at 4 °C, in DM at 37 °C, in BHI supplemented with acetic acid at 4 °C, in DM supplemented with ethanol at 37 °C, and in BHI supplemented with citric acid at 4 °C; Figure S2: Electron micrographs of *Listeria monocytogenes* EGD−e wild−type and *ΔsigB, ΔsigL*, and *ΔsigBL* mutant strains grown in BHI at 37 and 3 °C to mid−logarithmic growth phase, fixed with 5% glutaraldehyde, applied on carbon−coated grids, negatively stained with 1% phosphotungstic acid hydrate, and examined under a transmission electron microscope; Table S1: Bacterial strains, plasmids, and oligonucleotides used in this study; Table S2: Results from the BIOLOG phenotypic microarray comparative analysis of the *Listeria monocytogenes* ∆*sigB* and parental strains; Table S3: Results from the BIOLOG phenotypic microarray comparative analysis of the *Listeria monocytogenes ∆sigBL* and parental strains; Table S4: BIOLOG PM phenotypes detected in *Listeria monocytogenes ∆sigBL* but not in *∆sigB* and *∆sigL* strains; Table S5: Genes down−regulated in *Listeria monocytogenes ∆sigB* during exponential growth in BHI at 3 °C; Table S6: Genes down−regulated in *Listeria monocytogenes ∆sigB* during exponential growth in BHI at 37 °C; Table S7: Genes down−regulated in *Listeria monocytogenes ∆sigBL* during exponential growth in BHI at 3 °C; Table S8: Genes down−regulated in *Listeria monocytogenes ∆sigBL* during exponential growth in BHI at 37 °C; Table S9: Genes down−regulated in *Listeria monocytogenes ∆sigBL* but not in *∆sigB* or *∆sigL* during exponential growth in BHI at 3 °C; Table S10: Genes down−regulated in *Listeria monocytogenes ∆sigBL* but not in *∆sigB* or *∆sigL* during exponential growth in BHI at 37 °C.
