# Supplementary material for: Transcriptomic and Phenotypic Analyses of the Sigma B-Dependent Characteristics and the Synergism between Sigma B and Sigma L in Listeria monocytogenes EGD-e
Source: Microorganisms. 2020 Oct 23;8(11):1644. doi: 10.3390/microorganisms8111644 (PMC7690807; doi:10.3390/microorganisms8111644)
Supplement: Supplementary file 1 [file microorganisms-08-01644-s001.zip › microorganisms-964631--S/Figure S1_corrected.docx]

B

DM 4°C

A

BHI 4°C

D

DM 37°C

C

BHI 4°C plus

2.5% lactic acid, pH 6.0

E

BHI 4°C plus

0.35% acetic acid, pH 5.5

F

DM 37°C

plus 5% ethanol

G

BHI 4°C plus

0.35% citric acid,

pH 5.5

*L. monocytogenes* EGD-e

*∆sigB*

*∆sigBL*

**Figure S1.** Growth of *Listeria monocytogenes* EGD-e wild-type strain and *∆sigB* and *∆sigBL* mutant strains in BHI broth at 4°C (A), in DM at 4°C (B), in BHI supplemented with lactic acid at 4°C (C), in DM at 37 °C (D), in BHI supplemented with acetic acid at 4°C (E), in DM supplemented with ethanol at 37°C (F) and in BHI supplemented with citric acid at 4°C (G). The means and standard deviations derived from four independent experiments are presented.
