# Supplementary material for: Transcriptomic and Phenotypic Analyses of the Sigma B-Dependent Characteristics and the Synergism between Sigma B and Sigma L in Listeria monocytogenes EGD-e
Source: Microorganisms. 2020 Oct 23;8(11):1644. doi: 10.3390/microorganisms8111644 (PMC7690807; doi:10.3390/microorganisms8111644)
Supplement: Supplementary file 1 [file microorganisms-08-01644-s001.zip › microorganisms-964631--S/Figure S2.docx]

A B







EGD-e




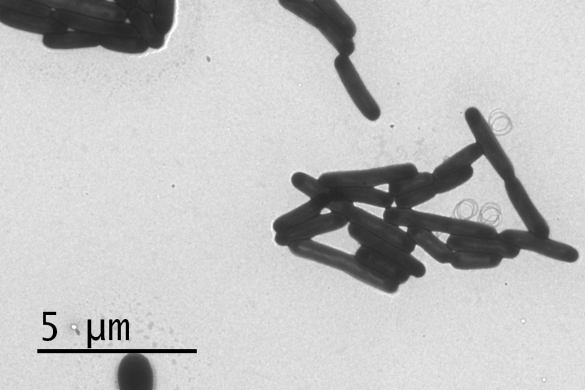


*ΔsigB*







*ΔsigL*







*ΔsigBL*

**Figure S2.** Electron micrographs of *Listeria monocytogenes* EGD-e wild-type and *ΔsigB, ΔsigL* and *ΔsigBL* mutant strains grown in BHI at 37°C (A) and 3°C (B) to mid-logarithmic growth phase, fixed with 5% glutaraldehyde, applied on carbon-coated grids, negatively stained with 1% phosphotungstic acid hydrate and examined under transmission electron microscope. Arrows indicate the presence of flagella.
