# Supplementary material for: Transcriptomic and Phenotypic Analyses of the Sigma B-Dependent Characteristics and the Synergism between Sigma B and Sigma L in Listeria monocytogenes EGD-e
Source: Microorganisms. 2020 Oct 23;8(11):1644. doi: 10.3390/microorganisms8111644 (PMC7690807; doi:10.3390/microorganisms8111644)
Supplement: Supplementary file 1 [file microorganisms-08-01644-s001.zip › microorganisms-964631--S/Table S10_corrected.docx]

**Table S10.** Genes down-regulated in *Listeria monocytogenes* *∆sigBL* but not in *∆sigB* or *∆sigL^a^* during exponential growth in BHI at 37°C^b^.

| **Gene** | **Functional category and protein^c^** |
| --- | --- |
| *lmo1628*  *lmo0629*  *lmo2101*  *lmo2463*  *lmo0321*  *lmo0433*  *lmo0434*  *lmo0110*  *lmo0244*  *lmo0655*  *lmo1911*  *lmo0246*  *lmo2194*  *lmo2353*  *lmo1656*  *lmo1753*  *lmo1776*  *lmo2387* | **Amino acid biosynthesis**  tryptophan synthase, beta subunit  **Biosynthesis of cofactors, prosthetic groups, and carriers**  isochorismatase family protein, putative  pyridoxine biosynthesis protein  **Cell envelope**  membrane protein, MmpL family, putative  **Cellular processes**  Heat-labile enterotoxin alpha chain domain protein  internalin A  internalin B  **Fatty acid and phospholipid metabolism**  lipase/esterase, putative  **Protein synthesis**  ribosomal protein L33  **Regulatory functions**  serine/threonine protein phosphatase  regulatory components of sensory transduction system    **Transcription**  transcription antitermination protein NusG  **Transport and binding proteins**  peptide ABC transporter, permease protein  Na+/H+ antiporter, putative  **Hypothetical and unclassified proteins**  hypothetical protein  conserved hypothetical protein  conserved hypothetical protein  conserved hypothetical protein |

^a^ [1]

^b^ The genes listed are those displaying ≥ 2.5 –fold (equivalent to ±1.3 log_2_) change (rounded to one digit) in transcript level between the *∆sigB, ∆sigL* and *∆sigBL* and the parental EGD-e strain with a moderated t-test statistical significance of P-value≤0.01.

^c^ Gene functional categories are based on the annotations provided by the Comprehensive Microbial Resource of the J. Craig Venter Institute (CMR-JCVI) (<http://cmr.jcvi.org>).

References

1. Mattila, M.; Somervuo, P.; Rattei, T.; Korkeala, H.; Stephan, R.; Tasara, T. Phenotypic and transcriptomic analyses of Sigma L−dependent characteristics in Listeria monocytogenes EGD−e. *Food Microbiol.* **2012**, *32*, 152–164
