# Supplementary material for: Transcriptomic and Phenotypic Analyses of the Sigma B-Dependent Characteristics and the Synergism between Sigma B and Sigma L in Listeria monocytogenes EGD-e
Source: Microorganisms. 2020 Oct 23;8(11):1644. doi: 10.3390/microorganisms8111644 (PMC7690807; doi:10.3390/microorganisms8111644)
Supplement: Supplementary file 1 [file microorganisms-08-01644-s001.zip › microorganisms-964631--S/Table S1_corrected.docx]

| **Table S1.**  Bacterial strains, plasmids, and oligonucleotides used in this study | | |
| --- | --- | --- |
| Materials | Description | References |
| **Bacterial strains** |  |  |
| *Listeria monocytogenes* |  |  |
| EGD-e | Wild-type serotype 1/2a strain | [1] |
| *ΔsigL* | 1290 bp in-frame deletion of the *sigL* gene | [2] |
| *ΔsigB* | 726 bp in-frame deletion of the *sigB* gene | This study |
| *ΔsigBL* | 726 and 1290 bp in-frame deletion of the *sigB* and *sigL* genes | This study |
| *Escherichia coli* |  |  |
| XL-1 Blue | Wild-type laboratory strain for routine plasmid propagation and cloning applications | [3] |
| **Plasmids** |  |  |
| pKSV7 | Temperature-sensitive Gram-positive bacteria integrational vector | [4] |
| **Oligonucleotides** |  |  |
| *Slicing-by-overlap extension (SOE) primers* | | |
| SOE-P-sigB-A^a^ | CCGGAATTCCAGCGCCAAAGGTAAAAGAAGCA | This study |
| SOE-P-sigB-B^b^ | *GCAACGCCTCTCGAAGTTGAGATACTTTTGG*CATTCTCCTC | This study |
| SOE-P-sigB-C^b^ | *CCAAAAGTATCTCAACTTCGAGAGGCGTTGC*AGAAT | This study |
| SOE-P-sigB-D^c^ | TCCCCCGGG TTCCGGAAATTTCCCAACCATAAAAG | This study |
| SOE-P-rpoN-A^a^ | CGGGATCCACCGCGCAGGACGCGT | [5] |
| SOE-P- rpoN-B^d^ | *CGTTTAGAATCTAATAATATTCCTTCTTCC*TCTAAAAGAAAAAGAT | [5] |
| SOE-P- rpoN-C^d^ | *AGGAAGAAGGATATTATTAGATTCTAAACG* CACATTAAAACCTC | [5] |
| SOE-P- rpoN-D^a^ | GGAATTCCAGGACTAACTCGCTTCGGA | [5] |
| *Real-time PCR primers* |  |  |
| *16S rRNA* forward | GATGCATAGCCGACCTGAGA | [2] |
| *16S rRNA* reverse | CTCCGTCAGACTTTCGTCCA | [2] |
| *lmo0096* forward | GATTCACGTCTCTTGCATGGT | [2] |
| *lmo0096* reverse | CTGGTGGTGCAGCTTGTTC | [2] |
| *lmo0137* forward | ACACGAGAGCGGAGTTTTTG | [2] |
| *lmo0137* reverse | AGGGTCATAAGGCGAAAGGA | [2] |
| *lmo0685* forward | cgtgctttggacaccattt | [2] |
| *lmo0685* reverse | tctcgttttcttcccttttcc | [2] |
| *lmo2625* forward | TCGGATGGGTAAAGGTAAAGG | [2] |
| *lmo2625* reverse | AGTTTTGACCGGCAGTTTGT | [2] |
|  |  |  |
|  |  |  |

^a^The *Eco*RI recognition sequence incorporated in primer to facilitate cloning is underlined.

^b^The complementary overhang regions in SOE-P-SigB-B and SOE-P-SigB-C SOE PCR primers are in italics.

^c^ The *Sma*I recognition sequence incorporated in primer to facilitate cloning is underlined.

^d^The complementary overhang regions in SOE-P-SigL-B and SOE-P-SigL-C SOE PCR primers are in italics.

**References:**

1. Glaser, P.; Frangeul, L., Buchrieser, C.; Rusniok, C.; Amend, A.; Baquero, F.; Berche, P.; Bloecker, H.; Brandt, P.; Chakraborty, T.; et al. Comparative genomics of Listeria species. *Science* **2001**, *294*, 849–852.

2. Mattila, M.; Somervuo, P.; Rattei, T.; Korkeala, H.; Stephan, R.; Tasara, T. Phenotypic and transcriptomic analyses of Sigma L−dependent characteristics in Listeria monocytogenes EGD−e. *Food Microbiol.* **2012**, *32*, 152–164.

3. Bullock, W.O. XL1−Blue: A high efficiency plasmid transforming recA Escherichia coli strain with beta−galactosidase selection. Bio. Tecchniques 1987, 5, 376–379.

4. Smith, K.; Youngman, P. Use of a new integrational vector to investigate compartment−specific expression of the Bacillus subtilis spoIIM gene. *Biochimie* **1992**, *74*, 705–711.

5. Raimann, E.; Schmid, B.; Stephan, R.; Tasara, T. The alternative sigma factor Sigma(L) of L. monocytogenes promotes growth under diverse environmental stresses. Foodborne Pathog. Dis. 2009, 6, 583–591
