# Supplementary material for: Transcriptomic and Phenotypic Analyses of the Sigma B-Dependent Characteristics and the Synergism between Sigma B and Sigma L in Listeria monocytogenes EGD-e
Source: Microorganisms. 2020 Oct 23;8(11):1644. doi: 10.3390/microorganisms8111644 (PMC7690807; doi:10.3390/microorganisms8111644)
Supplement: Supplementary file 1 [file microorganisms-08-01644-s001.zip › microorganisms-964631--S/Table S2_corrected.docx]

**Table S2.** Results from the BIOLOG phenotypic microarray comparative analysis of the *Listeria monocytogenes* ∆*sigB* and parental EGD-e strains

| **Phenotypic microarray test** | **Chemical/Substrate** | **Mode of action** | **Phenotype of the ∆*sigB*^a^** |
| --- | --- | --- | --- |
| **Chemical sensitivity (PM11-20)**  Phenotypes lost - slower growth/increased sensitivity | 2,4-Diamino-6,7-Diisopropylpteridine  Sanguinarine  Furaltadone  Chlortetracycline  Sodium bromate  Sodium periodate  Sodium Orthovanadate | ATPase, Na+/K+ and Mg++  DNA synthesis, nitro-compound, multiple sites  Protein synthesis; 30S ribosomal subunit; tetracycline  Toxic anion  Toxic anion, oxidizing agent  Transport, toxic anion, PO4 analog | -126  -80  -125  -93  -106  -104  -160 |

^a^Based on time-course curves of respiration (tetrazolium colour formation) rate. The differences in the curve areas of the mutant and the wild-type strains are calculated. The units are arbitrary. Negative values indicate that the parental strain showed higher rates of respiration than the mutant. Results are based on averages from two biological replicates.
