# Supplementary material for: Transcriptomic and Phenotypic Analyses of the Sigma B-Dependent Characteristics and the Synergism between Sigma B and Sigma L in Listeria monocytogenes EGD-e
Source: Microorganisms. 2020 Oct 23;8(11):1644. doi: 10.3390/microorganisms8111644 (PMC7690807; doi:10.3390/microorganisms8111644)
Supplement: Supplementary file 1 [file microorganisms-08-01644-s001.zip › microorganisms-964631--S/Table S3_corrected.docx]

**Table S3.** Results from the BIOLOG phenotypic microarray comparative analysis of the *Listeria monocytogenes* ∆*sigBL* and parental EGD-e strains

| **Phenotypic microarray test** | **Chemical/Substrate** | **Mode of action** | **Phenotype of the ∆*sigBL*^a^** |
| --- | --- | --- | --- |
| **Metabolism (PM01)**  Phenotypes lost - slower growth/ increased sensitivity  **Metabolism (PM02)**  Phenotypes lost - slower growth/ increased sensitivity  **Osmotic sensitivity and pH (PM09-PM10)**  Phenotypes lost – slower growth/ increased sensitivity  **Chemical sensitivity (PM11-20)**  Phenotypes lost - slower growth/increased sensitivity | N-Acetyl-D-Glucosamine  3-0-b-D-Galactopyranosyl-D-Arabinose  3% Sodium Formate  7% Urea  100mM Sodium Nitrite  2,4-Diamino-6,7-Diisopropylpteridine  Carbenicillin  Atropine  Ketoprofen  Patulin  Sanguinarine  Triclosan  Semicarbazide hydrochloride  Trifluoperazine  Sodium pyrophosphate decahydrate  EGTA  1,10-Phenanthroline  EDTA  1-Hydroxy-Pyridine-2-thione  5,7-Dichloro-8-hydroxyquinoline  5,7-Dichloro-8-hydroxy-quinaldine  Fusaric Acid  8-Hydroxyquinoline  Promethazine  Nitrofurazone  Phleomycin  4-Hydroxycoumarin  9-Aminoacridine  Acriflavine  2- Phenylphenol  Hexaminecobalt (III) Chloride  Furaltadone  Nitrofurantoin  Bleomycin  Ciprofloxacin  Norfloxacin  Pipemidic Acid  Enoxacin  Sulfisoxazole  Chloroxylenol  Oxycarboxin  Dodine  Aminotriazole  Procaine  Nordihydroguaiaretic acid  Guanidine hydrochloride  Poly-L-lysine  Domiphen bromide  Lauryl sulfobetaine  Colistin  Tinidazole  6-Mercaptopurine  Azathioprine  3% Sodium Formate  7% Urea  1-Chloro-2,4-Dinitrobenzene  Iodoacetate  Methyl viologen  3, 4-Dimethoxybenzyl alcohol  Compound 48/80  Chelerythrine  Chloramphenicol  Capreomycin  Chloramphenicol  Thiamphenicol  Spectinomycin  Blasticidin S  Vancomycin  Puromycin  Oleandomycin  Tobramycin  Sisomicin  Streptomycin  Paromycin  Oxytetracycline  Doxycycline  Penimepicycline  Tetracycline  Amikacin  Neomycin  Kanamycin  Chlortetracycline  Demeclocyline  Minocycline  5-Fluoro-5'-deoxyuridine  Crystal Violet  18-Crown-6-Ether  Sorbic acid  Pentachlorophenol (PCP)  Cinnamic acid  Sodium azide  3,5- Diamino-1,2,4-triazole (Guanazole)  2-Nitroimidazole  Hydroxyurea  Trifluorothymidine  Sodium bromate  Sodium m-arsenite  Sodium metasilicate  Sodium periodate  Antimony (III) chloride  100mM Sodium Nitrite  Boric Acid  Sodium Metaborate  Sodium Cyanate  Sodium Nitrite  Sodium Orthovanadate  Cadmium Chloride  Ferric Chloride  Lithium Chloride  L-Glutamic-g-Hydroxamate  Glycine Hydroxamate  Cefoxitin  Cefmetazole  Carbenicillin  Phenethicillin  Oxacillin  Penicillin G  Piperacillin  D-Cycloserine  Cefazolin  Cephalothin  Ceftriaxone  Amoxicillin  Cloxacillin  Nafcillin | Carbon source  Carbon source  Osmotic sensitivity, sodium formate  Osmotic sensitivity, urea  Toxicity, nitrite  Acetylcholine receptor, antagonist  Anti-capsule  Antifungal, tubulin binding  ATPase, Na+/K+ and Mg++  Bacterial fatty acid synthesis, enoyl-acyl carrier protein reductase  Carbonyl agent, semicarbazide-sensitive amine oxidase, DNA damage  Cell cycle modulation, DNA synthesis, Ca(2+)/ calmodulin dependent protein phosphorylation and lipid  Chelating agent  Chelator, Ca++  Chelator, Fe++, Zn++, divalent metal  Chelator, hydrophilic  Chelator, lipophilic  Chelator, lipophilic  Chelator, lipophilic  Chelator, lipophilic  Chelator, lipophilic  Cyclic nucleotide phosphodiesterase  DNA damage, multiple sites, nitrofuran analog DNA damage, oxidative, ionizing ratiation  DNA intercalator  DNA intercalator  DNA intercalator  DNA intercalator  DNA synthesis  DNA synthesis, nitro-compound, multiple sites DNA synthesis, nitro-compound, multiple sites DNA synthesis; polymerase inhibitor  DNA topoisomerase, quinolone  DNA topoisomerase, quinolone  DNA unwinding, gyrase (GN), topoisomerase (GP), quinolone  DNA unwinding; gyrase (GN); topoisomerase (GP); fluoroquinolone  Folate synthesis, PABA analog  Fungicide  Fungicide, carboxamide, respiratory enzymes Fungicide, guanidine, membrane permeability  Histidine biosynthesis, catalase  Ion channal inhibitor, Na+ (m)  Lipoxygenase, fungicide  Membrane, chaotropic agent  Membrane, detergent, cationic  Membrane, detergent, cationic, fungiside  Membrane, detergent, zwitterionic  Membrane; transport  Mutagen, nitroimidazole (GP, GN)  Nucleic acid analog, purine  Nucleic acid analog, purine  Osmotic sensitivity, sodium formate  Osmotic sensitivity, urea  Oxidation, glutathione  Oxidation, sulfhydryl  Oxidizing agent  Oxidizing agent, free radical-peroxidase substrate  Phospholipase C, ADP ribosylation  Protein kinase C  Protein synthesis  Protein synthesis  Protein synthesis  Protein synthesis  Protein synthesis  Protein synthesis  Protein synthesis  Protein synthesis, 30S ribosomal subunit, premature chanin termination  Protein synthesis, 50S ribosomal subunit, macrolide  Protein synthesis, aminoglycoside  Protein synthesis, aminoglycoside  Protein synthesis, aminoglycoside  Protein synthesis, aminoglycoside  Protein synthesis, tetracycline  Protein synthesis, tetracycline  Protein synthesis, tetracycline  Protein synthesis, tetracycline  Protein synthesis; 30S ribosomal subunit; aminoglycoside  Protein synthesis; 30S ribosomal subunit; aminoglycoside  Protein synthesis; 30S ribosomal subunit; aminoglycoside  Protein synthesis; 30S ribosomal subunit; tetracycline  Protein synthesis; 30S ribosomal subunit; tetracycline  Protein synthesis; 30S ribosomal subunit; tetracycline  Pyrimidine antimetabolite: inhibits nucleic acid replication  Respiration  Respiration, ionophore  Respiration, ionophore, H+  Respiration, ionophore, H+  Respiration, ionophore, H+  Respiration, uncoupler  Ribonucleotide DP reductase  Ribonucleotide DP reductase  Ribonucleotide DP reductase, antifolate (inhibits thymine and methionine synthesis)  Thymidylate synthetase, DNA polymerase  Toxic anion  Toxic anion  Toxic anion  Toxic anion, oxidizing agent  Toxic cation  Toxicity, nitrite  Transport, toxic anion  Transport, toxic anion  Transport, toxic anion  Transport, toxic anion  Transport, toxic anion, PO4 analog  Transport, toxic cation  Transport, toxic cation  Transport, toxic cation  tRNA synthetase  tRNA synthetase  Cell wall, cephalosporin  Cell wall, cephalosporin second generation  Cell wall, lactam  Cell wall, lactam  Cell wall, lactam  Cell wall, lactam  Cell wall, lactam  Cell wall, sphingolipid synthesis  Cell wall; cephalosporin first generation  Cell wall; cephalosporin first generation  Cell wall; cephalosporin third generation  Cell wall, lactam  Cell wall, lactam  Cell wall, lactam | -80  -60  -73  -93  -88  -240  -147  -217  -219  -120  -321  -351  -213  -93  -265  -315  -267  -370  -364  -332  -322  -240  -222  -318  -356  -245  -233  -231  -230  -167  -124  -376  -266  -288  -188  -85  -406  -213  -400  -163  -112  -52  -132  -344  -366  -264  -323  -135  -80  -257  -297  -330  -326  -73  -93  -100  -87  -262  -391  -189  -315  -360  -308  -257  -248  -239  -65  -63  -184  -186  -256  -197  -159  -128  -154  -123  -90  -89  -298  -194  -73  -247  -237  -137  -322  -113  -146  -95  -74  -68  -121  -399  -305  -117  -331  -377  -253  -231  -215  -185  -88  -352  -186  -98  -95  -235  -472  -126  -74  -171  -105  -330  -357  -346  -305  -292  -262  -234  -289  -335  -333  -275  -393  -346  -283 |

^a^Based on time-course curves of respiration (tetrazolium colour formation) rate. The differences in the curve areas of the mutant and the wild-type strains are calculated. The units are arbitrary. Negative values indicate that the parental strain showed higher rates of respiration than the mutant. Results are based on averages from two biological replicates.
