# Supplementary material for: Transcriptomic and Phenotypic Analyses of the Sigma B-Dependent Characteristics and the Synergism between Sigma B and Sigma L in Listeria monocytogenes EGD-e
Source: Microorganisms. 2020 Oct 23;8(11):1644. doi: 10.3390/microorganisms8111644 (PMC7690807; doi:10.3390/microorganisms8111644)
Supplement: Supplementary file 1 [file microorganisms-08-01644-s001.zip › microorganisms-964631--S/Table S4_corrected.docx]

**Table S4.** BIOLOG PM phenotypes detected in *Listeria monocytogenes* ∆*sigBL* but not in Δ*sigB* and Δ*sigL* strains

| **Phenotypic microarray test** | **Chemical/Substrate** | **Mode of action** | **Phenotype of the EGDe ∆*sigBL*^b^** |
| --- | --- | --- | --- |
| **Metabolism (PM02)**  Phenotypes lost - slower growth/ increased sensitivity  **Osmotic sensitivity and pH (PM09-PM10)**  Phenotypes lost – slower growth/ increased sensitivity  **Chemical sensitivity (PM11-20)**  Phenotypes lost - slower growth/increased sensitivity | 3-0-b-D-Galactopyranosyl-D-Arabinose  100mM Sodium Nitrite  Semicarbazide hydrochloride  Fusaric acid  4-Hydroxycoumarin  Enoxacin  Chloroxylenol  Lauryl sulfobetaine  Colistin  1-Chloro-2,4-Dinitrobenzene  Iodoacetate  Blasticidin S  Vancomycin  Sorbic acid  2-Nitroimidazole  Hydroxyurea  Ferric Chloride  L-Glutamic-g-Hydroxamate  Glycine Hydroxamate  Cefoxitin  Phenethicillin  D-Cycloserine  Ceftriaxone  Nafcillin | Carbon source  Toxicity, nitrite  DNA damage, carbonyl agent  Chelator, lipophilic  DNA intercalator  DNA unwinding, gyrase  Fungicide  Membrane, detergent, zwitterionic  Membrane, transport  Oxidation, glutathione  Oxidation, sulfhydryl  Protein synthesis  Protein synthesis  Respiration, ionophore, H+  Ribonucleotide DP reductase  Ribonucleotide DP reductase  Transport, toxic cation  tRNA synthetase  tRNA synthetase  Cell wall, cephalosporin  Cell wall, lactam  Cell wall, sphingolipid synthesis  Cell wall, cephalosporin third generation  Cell wall, lactam | -60  -88  -213  -240  -233  -213  -153  -80  -257  -100  -87  -65  -63  -95  -305  -117  -126  -171  -105  -330  -305  -289  -275  -283 |
|  |  |  |  |

^a^ [1]

^b^Based on time-course curves of respiration (tetrazolium colour formation) rate. The differences in the curve areas of the mutant and the parental strains are calculated. The units are arbitrary. Negative values indicate that the parental strain showed higher rates of respiration than the mutant. Results are based on averages from two biological replicates.

**Reference**

1. Mattila, M.; Somervuo, P.; Rattei, T.; Korkeala, H.; Stephan, R.; Tasara, T. Phenotypic and transcriptomic analyses of Sigma L−dependent characteristics in Listeria monocytogenes EGD−e. *Food Microbiol.* **2012**, *32*, 152–164.
