# Supplementary material for: Transcriptomic and Phenotypic Analyses of the Sigma B-Dependent Characteristics and the Synergism between Sigma B and Sigma L in Listeria monocytogenes EGD-e
Source: Microorganisms. 2020 Oct 23;8(11):1644. doi: 10.3390/microorganisms8111644 (PMC7690807; doi:10.3390/microorganisms8111644)
Supplement: Supplementary file 1 [file microorganisms-08-01644-s001.zip › microorganisms-964631--S/Table S5_corrected.docx]

**Table S5.** Genes down-regulated in *Listeria monocytogenes* *∆sigB* during exponential growth in BHI at 3°C^a^.

| **Gene** | **Functional category and protein^b^** |
| --- | --- |
| *lmo2001*  *lmo0781*  *lmo2002*  *lmo1046*  *lmo1043*  *lmo1047*  *lmo1048*  *lmo1045*  *lmo1042*  *lmo1044*  *lmo2278*  *lmo1079*  *lmo1285*  *lmo2291*  *lmo0433*  *lmo2230*  *lmo1580*  *lmo0263*  *lmo0515*  *lmo0690*  *lmo2673*  *lmo0723*  *lmo1879*  *lmo0321*  *lmo1601*  *lmo0669*  *lmo1967*  *lmo1439*  *lmo2016*  *lmo2217*  *lmo0711*  *lmo2426*  *lmo1694*  *lmo0712*  *lmo1288*  *lmo1577*  *lmo0134*  *lmo2700*  *lmo2370*  *lmo0078*  *lmo2434*  *lmo1726*  *lmo2719*  *lmo1256*  *lmo1561*  *lmo1368*  *lmo2743*  *lmo2584*  *lmo0913*  *lmo1406*  *lmo0722*  *lmo1917*  *lmo1579*  *lmo0813*  *lmo2674*  *lmo0502*  *lmo2341*  *lmo1667*  *lmo2425*  *lmo1992*  *lmo0210*  *lmo2695*  *lmo2696*  *lmo2288*  *lmo1381*  *lmo2279*  *lmo1407*  *lmo2157*  *lmo1097*  *lmo2280*  *lmo0211*  *lmo2511*  *lmo1463*  *lmo2085*  *lmo1172*  *lmo2583*  *lmo1173*  *lmo2582*  *lmo2792*  *lmo2173*  *lmo1189*  *lmo2739*  *lmo2714*  *lmo2329*  *lmo0200*  *lmo2334*  *lmo1367*  *lmo1102*  *lmo2003*  *lmo0649*  *lmo2422*  *lmo0483*  *lmo0887*  *lmo2004*  *lmo1150*  *lmo1295*  *lmo0895*  *lmo0784*  *lmo0783*  *lmo0782*  *lmo0169*  *lmo1964*  *lmo1100*  *lmo2581*  *lmo1041*  *lmo2297*  *lmo2296*  *lmo2292*  *lmo2285*  *lmo2299*  *lmo2293*  *lmo2284*  *lmo2283*  *lmo2286*  *lmo2281*  *lmo2289*  *lmo2330*  *lmo0717*  *lmo2158*  *lmo2067*  *lmo1112*  *lmo2269*  *lmo2742*  *lmo0265*  *lmo2294*  *lmo1115*  *lmo1966*  *lmo2423*  *lmo2740*  *lmo2300*  *lmo1104*  *lmo0754*  *lmo0800*  *lmo2295*  *lmo2298*  *lmo0111*  *lmo0966*  *lmo2287*  *lmo0674*  *lmo0625*  *lmo1111*  *lmo1230*  *lmo1049*  *lmo2216*  *lmo2174*  *lmo1912*  *lmo0602*  *lmo0596*  *lmo1099*  *lmo2697*  *lmo1113*  *lmo1602*  *lmo1241*  *lmo0995*  *lmo0170*  *lmo0019*  *lmo0724*  *lmo2568*  *lmo1109*  *lmo1229*  *lmo2301*  *lmo1114*  *lmo1108*  *lmo1107*  *lmo2748*  *lmo0964*  *lmo1105*  *lmo0670*  *lmo1106*  *lmo1612*  *lmo2585*  *lmo0965*  *lmo2213*  *lmo1668*  *lmo1334*  *lmo2104*  *lmo2643*  *lmo2340*  *lmo0955*  *lmo1103*  *lmo1333*  *lmo1098*  *lmo0994*  *lmo0471*  *lmo0937*  *lmo1190*  *lmo2258*  *lmo0654*  *lmo2454*  *lmo2567*  *lmo0954*  *lmo2156*  *lmo0718*  *lmo1597*  *lmo1791*  *lmo0673 lmo1110 lmo1380* | **Amino acid biosynthesis**  PTS system component  PTS system component  PTS system component  **Biosynthesis of cofactors, prosthetic groups, and carriers**  molybdenum cofactor biosynthesis protein MoaC  molybdopterin-guanine dinucleotide biosynthesis protein MobB  molybdopterin cofactor biosynthesis protein A  molybdenum cofactor biosynthesis protein B  molybdenum cofactor biosynthesis protein D/E  molybdopterin biosynthesis MoeA protein, putative  **Cell envelope**  molybdenum cofactor biosynthesis protein E  peptidoglycan lytic enzyme  membrane protein, putative  CoA binding domain protein  major tail shaft protein  **Cellular processes**  internalin A  arsenate reductase, putative  universal stress protein family  internalin H  universal stress protein family  flagellin  universal stress protein family  hemolysin secretion protein HylB, putative  cold-shock domain family protein-related protein  Heat-labile enterotoxin alpha chain domain protein  general stress protein  general stress protein 39  tellurite resistance protein  superoxide dismutase (mn)  cold-shock domain family protein-related protein  general stress protein, putative  flagellar basal-body rod protein FlgC  arsenate reductase, putative  cell division inhibitor  flagellar hook-basal body complex protein (FliE)  autoinducer-2 production protein LuxS  Metallo-beta-lactamase superfamily, putative  **Central intermediary metabolism**  acetyltransferase, GNAT family  oxidoreductase, aldo/keto reductase family  aminotransferase, class II  D-isomer specific 2-hydroxyacid dehydrogenase family protein  glutamate decarboxylase  oxidoreductase, Gfo/Idh/MocA family family  **DNA metabolism**  cytidine/deoxycytidylate deaminase family protein  MutT/nudix family protein, putative  replication initiation and membrane attachment protein, putative  DNA repair protein RecN  **Energy metabolism**  transaldolase, putative  formate dehydrogenase accessory protein FdhD  succinate-semialdehyde dehydrogenase  formate acetyltransferase  pyruvate oxidase  formate acetyltransferase  alanine dehydrogenase  fructokinase  ribose 5-phosphate isomerase B  KpsF/GutQ family protein, putative  carbohydrate kinase, PfkB family  L-lactate dehydrogenase  glycine cleavage system H protein  alpha-acetolactate decarboxylase  L-lactate dehydrogenase  dihydroxyacetone kinase family protein  dihydroxyacetone kinase family protein  gp15  **Fatty acid and phospholipid metabolism**  Acylphosphatase  **Mobile and extrachromosomal element functions**  holin  **Protein fate**  pyruvate formate-lyase activating enzyme  secretory protein (sepA)  integrase, phage family  gp23  **Protein synthesis**  ribosomal 5S rRNA E-loop binding protein Ctc/L25/TL5  ribosomal subunit interface protein  **Purines, pyrimidines, nucleosides, and nucleotides**  cytidine deaminase  **Regulatory functions**  Gram positive anchor domain protein  response regulator  DNA-binding response regulator  histidine kinase, putative  sensor histidine kinase  Helix-turn-helix domain protein  flagellar regulatory protein A  transcriptional regulator, AraC family  Sir2 family protein  Gram positive anchor domain protein  transcriptional regulator  listeriolysin regulatory protein  Helix-turn-helix domain protein  transcriptional regulator, ArgR family  arsenical resistance operon repressor, putative  transcriptional regulator, GntR family, putative  PUTATIVE TRANSCRIPTION REGULATOR PROTEIN, putative  DNA-binding response regulator  positive transcriptional activator, putative  Helix-turn-helix protein, copG family domain protein  transcriptional regulator, GntR family, putative  transcriptional regulator, AraC family, putative  host factor-I protein  **Transcription**  RNA polymerase sigma factor B  **Transport and binding proteins**  PTS system component  PTS system component  PTS system component  transporter, putative  ABC transporter, ATP-binding protein, putative  cation transport ATPase, E1-E2 family  permease, putative  molybdenum ABC transporter, periplasmic molybdate-binding protein  **Viral functions**  minor capsid protein  coat protein  gp11  gp18  putative portal protein  gp10  gp19  gp20  gp17  gp22-related protein  gp14  gp33  transglycosylase, putative  **Hypothetical and unclassified proteins**  conserved domain protein  choloylglycine hydrolase  FtsK/SpoIIIE family protein  YhzC  SH3 domain protein  peptidase, M20/M25/M40 family  gp9  LPXTG-motif cell wall anchor domain protein, putative  conserved domain protein, putative  AGR_C_4131p  cdd1  Rorf447  NLP/P60 family protein  PEP2, putative  HesB-like domain protein  gp8  gp4  Domain of unknown function 2, putative  TPR Domain domain protein  putative tape-measure protein  Rho-associated, coiled-coil forming protein kinase p160 ROCK-1, putative  Lipase/Acylhydrolase with GDSL-like motif domain protein  Helix-turn-helix domain protein  CvpA family protein  HesA/MoeB/ThiF family protein  HIT family protein  GGDEF family protein, putative  GGDEF family protein, putative  protease synthase and sporulation negative regulatory protein pai 1, putative  conserved hypothetical protein  conserved hypothetical protein  conserved hypothetical protein  conserved hypothetical protein  conserved hypothetical protein  conserved hypothetical protein  conserved hypothetical protein  conserved hypothetical protein  conserved hypothetical protein  conserved hypothetical protein  conserved hypothetical protein  conserved hypothetical protein  conserved hypothetical protein  conserved hypothetical protein  conserved hypothetical protein  conserved hypothetical protein  conserved hypothetical protein  conserved hypothetical protein  conserved hypothetical protein  conserved hypothetical protein  conserved hypothetical protein  conserved hypothetical protein  conserved hypothetical protein  conserved hypothetical protein  conserved hypothetical protein  conserved hypothetical protein  conserved hypothetical protein  conserved hypothetical protein  conserved hypothetical protein  conserved hypothetical protein  conserved hypothetical protein  conserved hypothetical protein  conserved hypothetical protein  conserved hypothetical protein  conserved hypothetical protein  hypothetical protein  hypothetical protein  hypothetical protein  hypothetical protein  hypothetical protein  hypothetical protein  hypothetical protein  hypothetical protein  hypothetical protein  hypothetical protein  hypothetical protein  hypothetical protein  hypothetical protein  hypothetical protein  hypothetical protein  hypothetical protein |

^a^ The log_2_-transformed mean fold change values were calculated from three biological replicates. Genes listed are those displaying ≥2.5 fold (equivalent to ±1.3 log_2_) change in transcript abundance between the *ΔsigB* and its parental EGD-e strain with a moderated t-test statistical significance of P-value ≤0.01.

^b^Gene functional categories are based on the annotations provided by the Comprehensive Microbial Resource of the J. Craig Venter Institute (CMR-JCVI) (http://cmr.jcvi.org).
