# Supplementary material for: Transcriptomic and Phenotypic Analyses of the Sigma B-Dependent Characteristics and the Synergism between Sigma B and Sigma L in Listeria monocytogenes EGD-e
Source: Microorganisms. 2020 Oct 23;8(11):1644. doi: 10.3390/microorganisms8111644 (PMC7690807; doi:10.3390/microorganisms8111644)
Supplement: Supplementary file 1 [file microorganisms-08-01644-s001.zip › microorganisms-964631--S/Table S6_corrected.docx]

**Table S6.** Genes down-regulated in *Listeria monocytogenes ∆sigB* during exponential growth in BHI at 37°C^a^

| **Gene** | **Functional category and protein^b^** |
| --- | --- |
| *lmo0223*  *lmo1627*  *lmo0096*  *lmo0097*  *lmo0098*  *lmo0781*  *lmo1551*  *lmo2571*  *lmo1433*  *lmo0880*  *lmo1079*  *lmo0263*  *lmo0421*  *lmo0515*  *lmo0669*  *lmo1694*  *lmo2230*  *lmo2673*  *lmo0152*  *lmo0134*  *lmo2434*  *lmo2573*  *lmo0554*  *lmo0043*  *lmo0539*  *lmo0722*  *lmo0913*  *lmo1634*  *lmo1883*  *lmo2674*  *lmo2724*  *lmo1407*  *lmo2157*  *lmo2398*  *lmo1885*  *lmo0445*  *lmo1298*  *lmo2085*  *lmo0895*  *lmo0135*  *lmo0136*  *lmo0137*  *lmo0169*  *lmo0524*  *lmo0782*  *lmo0783*  *lmo0784*  *lmo2192*  *lmo2715*  *lmo0265*  *lmo0355*  *lmo0593*  *lmo0912*  *lmo2067*  *lmo2158*  *lmo2602*  *lmo0602*  *lmo0099*  *lmo0121*  *lmo0133*  *lmo0170*  *lmo0596*  *lmo0642*  *lmo0670*  *lmo0794*  *lmo0796*  *lmo0903*  *lmo0911*  *lmo0995*  *lmo1140*  *lmo1241*  *lmo1261*  *lmo1830*  *lmo2213*  *lmo2391*  *lmo2570*  *lmo2572*  *lmo2603*  *lmo2748*  *lmo0628*  *lmo0654*  *lmo0937*  *lmo0953*  *lmo0994*  *lmo2132*  *lmo2454* | **Amino acid biosynthesis**  cysteine synthase A  tryptophan synthase, alpha subunit  PTS system component  PTS system component  PTS system component  PTS system component  **Biosynthesis of cofactors, prosthetic groups, and carriers**  folylpolyglutamate synthase  pyrazinamidase/nicotinamidase, putative  glutathione reductase, putative  **Cell envelope**  LPXTG-motif cell wall anchor domain protein, putative  membrane protein, putative  **Cellular processes**  internalin H  cell cycle family protein, putative  universal stress protein family  general stress protein 39  cell division inhibitor  arsenate reductase, putative  universal stress protein family  TraC protein  **Central intermediary metabolism**  acetyltransferase, GNAT family  glutamate decarboxylase  alcohol dehydrogenase, zinc-containing  iron-containing alcohol dehydrogenase  **Energy metabolism**  arginine deiminase  tagatose 1,6-diphosphate aldolase  pyruvate oxidase  succinate-semialdehyde dehydrogenase  aldehyde-alcohol dehydrogenase  chitinase, family 2  ribose 5-phosphate isomerase B  glyoxalase family protein  **Protein fate**  pyruvate formate-lyase activating enzyme  secretory protein (sepA)  low temperature requirement C protein  **Purines, pyrimidines, nucleosides, and nucleotides**  xanthine phosphoribosyltransferase  **Regulatory functions**  regulatory protein, putative  transcriptional regulator, MerR family  Gram positive anchor domain protein  **Transcription**  RNA polymerase sigma factor B  **Transport and binding proteins**  oligopeptide-binding protein appa precursor, putative  peptide ABC transporter, permease protein  oligopeptide transport permease protein appc  transporter, putative  sulfate transporter, putative  PTS system component  PTS system component  PTS system component  peptide ABC transporter, ATP-binding protein  transport ATP-binding protein CydC, putative  **Hypothetical and unclassified proteins**  peptidase, M20/M25/M40 family  succinate dehydrogenase/fumarate reductase, flavoprotein subunit  formate/nitrite transporter family protein  formate/nitrite transporter family protein  choloylglycine hydrolase  conserved domain protein  MgtC family protein  protease synthase and sporulation negative regulatory protein pai 1, putative  conserved hypothetical protein  conserved hypothetical protein  conserved hypothetical protein  conserved hypothetical protein  conserved hypothetical protein  conserved hypothetical protein  conserved hypothetical protein  conserved hypothetical protein  conserved hypothetical protein  conserved hypothetical protein  conserved hypothetical protein  conserved hypothetical protein  conserved hypothetical protein  conserved hypothetical protein  conserved hypothetical protein  conserved hypothetical protein  conserved hypothetical protein  conserved hypothetical protein  conserved hypothetical protein  conserved hypothetical protein  conserved hypothetical protein  conserved hypothetical protein  hypothetical protein  hypothetical protein  hypothetical protein  hypothetical protein  hypothetical protein  hypothetical protein  hypothetical protein |

^a^ The log_2_-transformed mean fold change values were calculated from three biological replicates. Genes listed are those displaying ≥2.5 fold (equivalent to ±1.3 log_2_) change in transcript abundance between the *ΔsigB* and its parental EGD-e strain with a moderated t-test statistical significance of P-value ≤0.01.

^b^ Functional category- Genes are functionally categorized based on the annotations

provided by the Comprehensive Microbial Resource of the J. Craig Venter Institute

(CMR-JCVI) (http://cmr.jcvi.org).
