# Supplementary material for: Transcriptomic and Phenotypic Analyses of the Sigma B-Dependent Characteristics and the Synergism between Sigma B and Sigma L in Listeria monocytogenes EGD-e
Source: Microorganisms. 2020 Oct 23;8(11):1644. doi: 10.3390/microorganisms8111644 (PMC7690807; doi:10.3390/microorganisms8111644)
Supplement: Supplementary file 1 [file microorganisms-08-01644-s001.zip › microorganisms-964631--S/Table S7_corrected.docx]

**Table S7.** Genes down-regulated in *Listeria monocytogenes* *∆sigBL* during exponential growth in BHI at 3°C^a^.

| **Gene** | **Functional category and protein^b^** |
| --- | --- |
| *lmo1986*  *lmo1990*  *lmo1988*  *lmo1989*  *lmo1154*  *lmo1985*  *lmo1984*  *lmo1987*  *lmo1991*  *lmo1983*  *lmo2006*  *lmo2545*  *lmo0561*  *lmo0781*  *lmo1195*  *lmo2211*  *lmo2571*  *lmo1672*  *lmo0584*  *lmo0688*  *lmo2754*  *lmo0433*  *lmo0515*  *lmo0690*  *lmo2230*  *lmo1580*  *lmo0263*  *lmo0689*  *lmo0723*  *lmo0601*  *lmo1694*  *lmo0669*  *lmo1439*  *lmo0711*  *lmo0712*  *lmo0710*  *lmo2673*  *lmo2738*  *lmo0152*  *lmo2569*  *lmo0134*  *lmo1736*  *lmo2017*  *lmo0158*  *lmo2434*  *lmo1155*  *lmo1287*  *lmo1286*  *lmo1574*  *lmo2702*  *lmo1634*  *lmo1293*  *lmo1917*  *lmo0210*  *lmo1538*  *lmo1406*  *lmo1166*  *lmo0913*  *lmo1163*  *lmo2674*  *lmo2467*  *lmo2455*  *lmo0016*  *lmo2341*  *lmo1936*  *lmo1992*  *lmo1571*  *lmo2456*  *lmo0788*  *lmo0232*  *lmo1161*  *lmo2201*  *lmo1165*  *lmo1572*  *lmo2202*  *lmo1407*  *lmo1666*  *lmo1375*  *lmo1393*  *lmo1578*  *lmo0251*  *lmo2511*  *lmo0250*  *lmo0211*  *lmo1480*  *lmo1218*  *lmo0248*  *lmo0249*  *lmo2620*  *lmo2624*  *lmo1660*  *lmo2615*  *lmo2618*  *lmo2625*  *lmo2617*  *lmo2621*  *lmo2623*  *lmo1816*  *lmo2616*  *lmo2619*  *lmo2626*  *lmo1330*  *lmo1540*  *lmo1783*  *lmo2448*  *lmo2614*  *lmo2622*  *lmo2627*  *lmo0046*  *lmo0237*  *lmo2605*  *lmo1797*  *lmo2613*  *lmo2653*  *lmo1764*  *lmo1766*  *lmo1765*  *lmo1767*  *lmo1768*  *lmo1769*  *lmo1770*  *lmo0279*  *lmo1772*  *lmo2611*  *lmo2085*  *lmo0402*  *lmo2173*  *lmo1517*  *lmo0200*  *lmo0926*  *lmo1102*  *lmo1280*  *lmo2792*  *lmo1956*  *lmo0651*  *lmo2088*  *lmo2461*  *lmo0895*  *lmo1327*  *lmo2449*  *lmo1848*  *lmo1849*  *lmo1539*  *lmo0398*  *lmo0399*  *lmo0135*  *lmo0782*  *lmo1732*  *lmo1738*  *lmo0783*  *lmo0784*  *lmo1167*  *lmo0400*  *lmo1739*  *lmo0136*  *lmo0923*  *lmo1845*  *lmo2087*  *lmo0925*  *lmo1516*  *lmo2382*  *lmo0137*  *lmo1391*  *lmo1731*  *lmo1740*  *lmo0283*  *lmo0284*  *lmo0798*  *lmo1425*  *lmo1426*  *lmo2383*  *lmo0169*  *lmo1431*  *lmo2381*  *lmo1847*  *lmo2158*  *lmo1115*  *lmo0401*  *lmo2269*  *lmo1112*  *lmo1153*  *lmo1158*  *lmo2067*  *lmo1159*  *lmo1162*  *lmo1160*  *lmo1152*  *lmo0265*  *lmo1156*  *lmo1104*  *lmo0757*  *lmo1164*  *lmo0216*  *lmo0355*  *lmo1796*  *lmo2029*  *lmo1970*  *lmo0760*  *lmo1157*  *lmo2423*  *lmo2433*  *lmo0562*  *lmo2411*  *lmo1111*  *lmo0602*  *lmo1067*  *lmo1479*  *lmo1110*  *lmo1099*  *lmo1113*  *lmo1114*  *lmo1776*  *lmo1107*  *lmo2340*  *lmo1106*  *lmo1109*  *lmo2748*  *lmo0724*  *lmo0995*  *lmo1105*  *lmo1108*  *lmo1526*  *lmo0796*  *lmo0911*  *lmo2705*  *lmo0170*  *lmo0596*  *lmo1750*  *lmo1771*  *lmo0525*  *lmo0572*  *lmo1098*  *lmo1103*  *lmo2213*  *lmo0730*  *lmo1241*  *lmo2572*  *lmo0019*  *lmo0600*  *lmo0666*  *lmo1637*  *lmo2669*  *lmo2567*  *lmo1257*  *lmo0994*  *lmo0937*  *lmo0471*  *lmo0687*  *lmo0718*  *lmo1190*  *lmo2454*  *lmo0393*  *lmo0654*  *lmo0715* | **Amino acid biosynthesis**  ketol-acid reductoisomerase  3-isopropylmalate dehydratase, small subunit  3-isopropylmalate dehydrogenase  3-isopropylmalate dehydratase, large subunit  adenosylcobalamin-dependent diol dehydratase beta subunit  acetolactate synthase, small subunit  acetolactate synthase, large subunit, biosynthetic type  2-isopropylmalate synthase  threonine dehydratase, biosynthetic  dihydroxy-acid dehydratase  acetolactate synthase  homoserine kinase  phosphoribosyl-ATP pyrophosphohydrolase  PTS system component  **Biosynthesis of cofactors, prosthetic groups, and carriers**  precorrin-6y c5,15-methyltransferase, putative  ferrochelatase  pyrazinamidase/nicotinamidase, putative  O-succinylbenzoic acid--CoA ligase, putative  **Cell envelope**  membrane protein  glycosyl transferase, group 2 family protein domain protein  D-alanyl-D-alanine carboxypeptidase  **Cellular processes**  internalin A  universal stress protein family  flagellin  arsenate reductase, putative  universal stress protein family  internalin H  chemotaxis protein CheV  hemolysin secretion protein HylB, putative  adhesin, putative  cell division inhibitor  general stress protein 39  superoxide dismutase (mn)  flagellar basal-body rod protein FlgC  flagellar hook-basal body complex protein (FliE)  flagellar basal-body rod protein FlgB  universal stress protein family  hemolysin  TraC protein  TraC protein  **Central intermediary metabolism**  acetyltransferase, GNAT family  acetyltransferase, putative  acid phosphatase, putative  hydrolase, haloacid dehalogenase-like family  glutamate decarboxylase  diol dehydrase (diol dehydratase) gamma subunit  **DNA metabolism**  DNA topoisomerase IV, A subunit  DNA topoisomerase IV, B subunit  DNA polymerase III, alpha subunit  recombination protein RecR  **Energy metabolism**  aldehyde-alcohol dehydrogenase  aerobic glycerol-3-phosphate dehydrogenase  formate acetyltransferase  L-lactate dehydrogenase  glycerol kinase  formate acetyltransferase  alcohol dehydrogenase, iron-containing  succinate-semialdehyde dehydrogenase  carbon dioxide concentrating mechanism protein  ribose 5-phosphate isomerase B  chitinase, putative  enolase  quinol oxidase, subunit IV  carbohydrate kinase, PfkB family  glycerol-3-phosphate dehydrogenase, NAD-dependent  alpha-acetolactate decarboxylase  Phosphofructokinase  phosphoglycerate mutase, 2,3-bisphosphoglycerate-independent  (R)-2-hydroxyglutaryl-CoA dehydratase activator-related protein  ClpC ATPase  **Fatty acid and phospholipid metabolism**  ethanolamine utilization protein eutj  3-oxoacyl-(acyl-carrier-protein) synthase II  aldehyde-alcohol dehydrogenase, putative  acetyl-CoA carboxylase, carboxyl transferase, alpha subunit  3-oxoacyl-(acyl-carrier-protein) synthase III  **Protein fate**  pyruvate formate-lyase activating enzyme  Peptidase family M1 domain protein  peptidase T, putative  peptidase, M16 family  proline dipeptidase  **Protein synthesis**  ribosomal protein L7/L12  ribosomal subunit interface protein  ribosomal protein L10  ribosomal 5S rRNA E-loop binding protein Ctc/L25/TL5  ribosomal protein S20  RNA methyltransferase, TrmH family  ribosomal protein L11  ribosomal protein L1  ribosomal protein L5  ribosomal protein L29  leucyl-tRNA synthetase  ribosomal protein S5  ribosomal protein S8  ribosomal protein L16  ribosomal protein L6  ribosomal protein L24  ribosomal protein S17  ribosomal protein L28  ribosomal protein L18  ribosomal protein S14p/S29e  ribosomal protein S3  ribosomal protein S15  ribosomal protein L27  ribosomal protein L20  SsrA-binding protein  ribosomal protein L30  ribosomal protein L14  ribosomal protein L22  ribosomal protein S18  glutamyl-tRNA synthetase  ribosomal protein L17  ribosomal protein S16  ribosomal protein L15  translation elongation factor Tu  **Purines, pyrimidines, nucleosides, and nucleotides**  phosphoribosylamine--glycine ligase  phosphoribosylglycinamide formyltransferase  phosphoribosylaminoimidazolecarboxamide formyltransferase/IMP cyclohydrolase  phosphoribosylformylglycinamidine cyclo-ligase  amidophosphoribosyltransferase  phosphoribosylformylglycinamidine synthase II  phosphoribosylformylglycinamidine synthetase I  anaerobic ribonucleoside-triphosphate reductase  phosphoribosylaminoimidazole-succinocarboxamide synthase  adenylate kinase  **Regulatory functions**  Gram positive anchor domain protein  transcriptional antiterminator, bglG family, putative  flagellar regulatory protein A  Nitrogen regulatory protein P-II  listeriolysin regulatory protein  transcriptional regulator, TetR family domain protein  arsenical resistance operon repressor, putative  dipeptide transport operon repressor  Helix-turn-helix domain protein  transcriptional regulator, Fur family  transcriptional regulator, gntR family domain protein  transcriptional regulator, TetR family  **Transcription**  Sigma-54 factors family  RNA polymerase sigma factor B  ribosome-binding factor A  ribonuclease R  **Transport and binding proteins**  iron (chelated) ABC transporter, permease protein  ABC transporter, ATP-binding protein  glycerol uptake facilitator protein  PTS system, fructose-specific IIABC component  PTS system, fructose-specific IIBC component  oligopeptide-binding protein appa precursor, putative  PTS system component  sugar ABC transporter, permease protein  amino acid ABC transporter, amino acid-binding protein  PTS system component  PTS system component  glycerol uptake facilitator protein  PTS system, fructose-specific IIBC component  amino acid ABC transporter, ATP-binding protein  peptide ABC transporter, permease protein  ABC transporter, ATP-binding protein, putative  xanthine/uracil permease family protein  MATE efflux family protein, putative  ABC transporter efflux protein, DrrB family, putative  ammonium transporter  Na+/H+ antiporter, MnhE component  oligopeptide transport permease protein appc  sugar ABC transporter, permease protein  sugar ABC transporter, permease protein  amino acid ABC transporter, permease protein  ABC transporter, permease protein  ABC transporter, ATP-binding protein  amino acid permease  amino acid ABC transporter, permease protein  osmoprotectant ABC transporter, permease protein  Na+/H+ antiporter, MnhF component  transporter, putative  ABC transporter, ATP-binding protein  Na+/H+ antiporter, MnhD component  **Hypothetical and unclassified proteins**  endocarditis specific antigen  conserved domain protein  LPXTG-motif cell wall anchor domain protein, putative  Glycosyl hydrolases family 38 domain protein  YhzC  FtsK/SpoIIIE family protein  diol dehydrase(diol dehydratase) alpha subunit  pduJ protein  choloylglycine hydrolase  pduJ protein  PduM, putative  PduL  pdub protein  peptidase, M20/M25/M40 family  PduG  NLP/P60 family protein  Conserved membrane protein, possible permease, YHCI B.subtilis ortholog, putative  DUF80 domain protein  S4 domain protein  succinate dehydrogenase/fumarate reductase, flavoprotein subunit  KH domain protein  YlmG protein  parathion hydrolase, putative  Phospholipase/Carboxylesterase superfamily  PduH  AGR_C_4131p  tributyrin esterase  phosphoribosyl-ATP pyrophosphatase/phosphoribosyl-AMP cyclohydrolase  Uncharacterized protein family (UPF0051) family  Helix-turn-helix domain protein  protease synthase and sporulation negative regulatory protein pai 1, putative  GTP-binding protein TypA  GTP-binding protein LepA  conserved hypothetical protein  conserved hypothetical protein  conserved hypothetical protein  conserved hypothetical protein  conserved hypothetical protein  conserved hypothetical protein  conserved hypothetical protein  conserved hypothetical protein  conserved hypothetical protein  conserved hypothetical protein  conserved hypothetical protein  conserved hypothetical protein  conserved hypothetical protein  conserved hypothetical protein  conserved hypothetical protein  conserved hypothetical protein  conserved hypothetical protein  conserved hypothetical protein  conserved hypothetical protein  conserved hypothetical protein  conserved hypothetical protein  conserved hypothetical protein  conserved hypothetical protein  conserved hypothetical protein  conserved hypothetical protein  conserved hypothetical protein  conserved hypothetical protein  conserved hypothetical protein  conserved hypothetical protein  conserved hypothetical protein  conserved hypothetical protein  conserved hypothetical protein  conserved hypothetical protein  conserved hypothetical protein  conserved hypothetical protein  hypothetical protein  hypothetical protein  hypothetical protein  hypothetical protein  hypothetical protein  hypothetical protein  hypothetical protein  hypothetical protein  hypothetical protein  hypothetical protein  hypothetical protein |

^a^ The log_2_-transformed mean fold change values were calculated from three biological replicates. Genes listed are those displaying ≥2.5 fold (equivalent to ±1.3 log_2_) change in transcript abundance between the *ΔsigBL* and its parental EGD-e strain with a moderated t-test statistical significance of P-value ≤0.01.

^b^ Functional category- Genes are functionally categorized based on the annotations

provided by the Comprehensive Microbial Resource of the J. Craig Venter Institute

(CMR-JCVI) (http://cmr.jcvi.org).
