# Supplementary material for: Transcriptomic and Phenotypic Analyses of the Sigma B-Dependent Characteristics and the Synergism between Sigma B and Sigma L in Listeria monocytogenes EGD-e
Source: Microorganisms. 2020 Oct 23;8(11):1644. doi: 10.3390/microorganisms8111644 (PMC7690807; doi:10.3390/microorganisms8111644)
Supplement: Supplementary file 1 [file microorganisms-08-01644-s001.zip › microorganisms-964631--S/Table S8_corrected.docx]

**Table S8.** Genes down-regulated in *Listeria monocytogenes* *∆sigBL* during exponential growth in BHI at 37°C^a^.

| **Gene** | **Functional category and protein^b^** |
| --- | --- |
| *lmo0223*  *lmo1299*  *lmo1627*  *lmo1628*  *lmo0096*  *lmo0097*  *lmo0098*  *lmo0781*  *lmo2685*  *lmo0629*  *lmo1551*  *lmo2101*  *lmo2571*  *lmo1433*  *lmo0880*  *lmo1079*  *lmo2463*  *lmo0263*  *lmo0321*  *lmo0433*  *lmo0434*  *lmo0515*  *lmo0669*  *lmo1694*  *lmo2230*  *lmo2507*  *lmo2673*  *lmo0152*  *lmo0134*  *lmo2434*  *lmo2573*  *lmo0554*  *lmo0043*  *lmo0210*  *lmo0539*  *lmo0722*  *lmo0913*  *lmo1634*  *lmo1883*  *lmo2457*  *lmo2674*  *lmo2717*  *lmo2724*  *lmo0110*  *lmo1407*  *lmo2157*  *lmo2612*  *lmo0244*  *lmo0250*  *lmo0251*  *lmo1783*  *lmo1785*  *lmo2605*  *lmo2610*  *lmo2613*  *lmo2614*  *lmo2615*  *lmo2616*  *lmo2617*  *lmo2618*  *lmo2619*  *lmo2620*  *lmo2621*  *lmo2622*  *lmo2624*  *lmo2625*  *lmo2626*  *lmo2633*  *lmo1885*  *lmo2611*  *lmo0445*  *lmo1298*  *lmo1911*  *lmo1956*  *lmo2085*  *lmo2460*  *lmo0655*  *lmo0246*  *lmo0895*  *lmo2461*  *lmo2606*  *lmo0135*  *lmo0136*  *lmo0137*  *lmo0169*  *lmo0524*  *lmo0641*  *lmo0782*  *lmo0783*  *lmo0784*  *lmo2192*  *lmo2193*  *lmo2194*  *lmo2353*  *lmo2715*  *lmo2716*  *lmo0265*  *lmo0355*  *lmo0593*  *lmo0912*  *lmo1651*  *lmo1652*  *lmo2067*  *lmo2102*  *lmo2158*  *lmo2602*  *lmo0602*  *lmo0099*  *lmo0170*  *lmo0439*  *lmo0596*  *lmo0642*  *lmo0670*  *lmo0794*  *lmo0903*  *lmo0911*  *lmo0995*  *lmo1140*  *lmo1241*  *lmo1261*  *lmo1753*  *lmo1776*  *lmo1830*  *lmo2048*  *lmo2213*  *lmo2387*  *lmo2391*  *lmo2570*  *lmo2572*  *lmo2603*  *lmo2748*  *lmo0628*  *lmo0654*  *lmo0937*  *lmo0953*  *lmo0994*  *lmo1656*  *lmo2132*  *lmo2454* | **Amino acid biosynthesis**  cysteine synthase A  glutamine synthetase, type I  tryptophan synthase, alpha subunit  tryptophan synthase, beta subunit  PTS system component  PTS system component  PTS system component  PTS system component  PTS system component  **Biosynthesis of cofactors, prosthetic groups, and carriers**  isochorismatase family protein, putative  folylpolyglutamate synthase  pyridoxine biosynthesis protein  pyrazinamidase/nicotinamidase, putative  glutathione reductase, putative  **Cell envelope**  LPXTG-motif cell wall anchor domain protein, putative  membrane protein, putative  membrane protein, MmpL family, putative  **Cellular processes**  internalin H  Heat-labile enterotoxin alpha chain domain protein  internalin A  internalin B  universal stress protein family  general stress protein 39  cell division inhibitor  arsenate reductase, putative  cell division ATP-binding protein FtsE  universal stress protein family  TraC protein  **Central intermediary metabolism**  acetyltransferase, GNAT family  glutamate decarboxylase  alcohol dehydrogenase, zinc-containing  iron-containing alcohol dehydrogenase  **Energy metabolism**  arginine deiminase  L-lactate dehydrogenase  tagatose 1,6-diphosphate aldolase  pyruvate oxidase  succinate-semialdehyde dehydrogenase  aldehyde-alcohol dehydrogenase  chitinase, family 2  triosephosphate isomerase  ribose 5-phosphate isomerase B  cytochrome d ubiquinol oxidase, subunit II  glyoxalase family protein  **Fatty acid and phospholipid metabolism**  lipase/esterase, putative  **Protein fate**  pyruvate formate-lyase activating enzyme  secretory protein (sepA)  preprotein translocase, SecY subunit  **Protein synthesis**  ribosomal protein L33  ribosomal protein L10  ribosomal protein L7/L12  ribosomal protein L20  translation initiation factor IF-3  ribosomal protein L17  translation initiation factor IF-1  ribosomal protein L15  ribosomal protein L30  ribosomal protein S5  ribosomal protein L18  ribosomal protein L6  ribosomal protein S8  ribosomal protein S14p/S29e  ribosomal protein L5  ribosomal protein L24  ribosomal protein L14  ribosomal protein L29  ribosomal protein L16  ribosomal protein S3  ribosomal protein S10  **Purines, pyrimidines, nucleosides, and nucleotides**  xanthine phosphoribosyltransferase  adenylate kinase  **Regulatory functions**  regulatory protein, putative  transcriptional regulator, MerR family  regulatory components of sensory transduction system  transcriptional regulator, Fur family  Gram positive anchor domain protein  gap transcriptional regulator  serine/threonine protein phosphatase  **Transcription**  transcription antitermination protein NusG  RNA polymerase sigma factor B  Sigma-54 factors family  DNA-directed RNA polymerase, alpha subunit  **Transport and binding proteins**  oligopeptide-binding protein appa precursor, putative  peptide ABC transporter, permease protein  oligopeptide transport permease protein appc  transporter, putative  sulfate transporter, putative  cation-transporting ATPase, E1-E2 family  PTS system component  PTS system component  PTS system component  peptide ABC transporter, ATP-binding protein  peptide ABC transporter, ATP-binding protein  peptide ABC transporter, permease protein  Na+/H+ antiporter, putative  transport ATP-binding protein CydC, putative  transport ATP-binding protein CydD, putative  **Hypothetical and unclassified proteins**  peptidase, M20/M25/M40 family  succinate dehydrogenase/fumarate reductase, flavoprotein subunit  formate/nitrite transporter family protein  formate/nitrite transporter family protein  ABC transporter, ATP-binding/permease protein  ABC transporter, ATP-binding/permease protein  choloylglycine hydrolase  Uncharacterized protein family UPF0030 superfamily  conserved domain protein  MgtC family protein  protease synthase and sporulation negative regulatory protein pai 1, putative  conserved hypothetical protein  conserved hypothetical protein  conserved hypothetical protein  conserved hypothetical protein  conserved hypothetical protein  conserved hypothetical protein  conserved hypothetical protein  conserved hypothetical protein  conserved hypothetical protein  conserved hypothetical protein  conserved hypothetical protein  conserved hypothetical protein  conserved hypothetical protein  conserved hypothetical protein TIGR00147  conserved hypothetical protein  conserved hypothetical protein  Uncharacterized ACR, COG1399  conserved hypothetical protein  conserved hypothetical protein  conserved hypothetical protein  conserved hypothetical protein  conserved hypothetical protein  conserved hypothetical protein  conserved hypothetical protein  hypothetical protein  hypothetical protein  hypothetical protein  hypothetical protein  hypothetical protein  hypothetical protein  hypothetical protein  hypothetical protein |

^a^ The log_2_-transformed mean fold change values were calculated from three biological replicates. Genes listed are those displaying ≥2.5 fold (equivalent to ±1.3 log_2_) change in transcript abundance between the *ΔsigBL* and its parental EGD-e strain with a moderated t-test statistical significance of P-value ≤0.01.

^b^ Functional category- Genes are functionally categorized based on the annotations

provided by the Comprehensive Microbial Resource of the J. Craig Venter Institute

(CMR-JCVI) (http://cmr.jcvi.org).
