# Supplementary material for: Transcriptomic and Phenotypic Analyses of the Sigma B-Dependent Characteristics and the Synergism between Sigma B and Sigma L in Listeria monocytogenes EGD-e
Source: Microorganisms. 2020 Oct 23;8(11):1644. doi: 10.3390/microorganisms8111644 (PMC7690807; doi:10.3390/microorganisms8111644)
Supplement: Supplementary file 1 [file microorganisms-08-01644-s001.zip › microorganisms-964631--S/Table S9_corrected.docx]

**Table S9.** Genes down-regulated in *Listeria monocytogenes* *∆sigBL* but not in *∆sigB* or *∆sigL^a^* during exponential growth in BHI at 3°C^b^.

| **Gene** | **Functional category and protein^c^** |
| --- | --- |
| *lmo1986*  *lmo1990*  *lmo1989*  *lmo1988*  *lmo1154*  *lmo1985*  *lmo1984*  *lmo1987*  *lmo1991*  *lmo1983*  *lmo2545*  *lmo0561*  *lmo1195*  *lmo2571*  *lmo2211*  *lmo1672*  *lmo0584*  *lmo2754*  *lmo2738*  *lmo0152*  *lmo2569*  *lmo1736*  *lmo2017*  *lmo0158*  *lmo2017*  *lmo1155*  *lmo1287*  *lmo1286*  *lmo1574*  *lmo2702*  *lmo1634*  *lmo1166*  *lmo1163*  *lmo2467*  *lmo2455*  *lmo1936*  *lmo2456*  *lmo1571*  *lmo0788*  *lmo1161*  *lmo2201*  *lmo1165*  *lmo1572*  *lmo2202*  *lmo1375*  *lmo1393*  *lmo1578*  *lmo0251*  *lmo0250*  *lmo1480*  *lmo1218*  *lmo2620*  *lmo2624*  *lmo0248*  *lmo0249*  *lmo2615*  *lmo2618*  *lmo1660*  *lmo2625*  *lmo2617*  *lmo2621*  *lmo2623*  *lmo2619*  *lmo2616*  *lmo2626*  *lmo1816*  *lmo2614*  *lmo2622*  *lmo2627*  *lmo1783*  *lmo1330*  *lmo1540*  *lmo2448*  *lmo0237*  *lmo2605*  *lmo0046*  *lmo2613*  *lmo2653*  *lmo1797*  *lmo1764*  *lmo1766*  *lmo1765*  *lmo1767*  *lmo1769*  *lmo1768*  *lmo1770*  *lmo0279*  *lmo2611*  *lmo1772*  *lmo0402*  *lmo1517*  *lmo0926*  *lmo1280*  *lmo1956*  *lmo0651*  *lmo2088*  *lmo1327*  *lmo2449*  *lmo1849*  *lmo0398*  *lmo0399*  *lmo0135*  *lmo1167*  *lmo0400*  *lmo0923*  *lmo2087*  *lmo0136*  *lmo1845*  *lmo0925*  *lmo2382*  *lmo0137*  *lmo1391*  *lmo0798*  *lmo0283*  *lmo2383*  *lmo1426*  *lmo0284*  *lmo1425*  *lmo2381*  *lmo1205*  *lmo1431*  *lmo1158*  *lmo1153*  *lmo1162*  *lmo1159*  *lmo1160*  *lmo1152*  *lmo1156*  *lmo1164*  *lmo0757*  *lmo0216*  *lmo0355*  *lmo2029*  *lmo1796*  *lmo0760*  *lmo1157*  *lmo2433*  *lmo0562*  *lmo2411*  *lmo1067*  *lmo1479*  *lmo1526*  *lmo0796*  *lmo0911*  *lmo2705*  *lmo1771*  *lmo1750*  *lmo0572*  *lmo0525*  *lmo0730*  *lmo2572*  *lmo1637*  *lmo0599*  *lmo2669*  *lmo0666*  *lmo1257*  *lmo0393* | **Amino acid biosynthesis**  ketol-acid reductoisomerase  3-isopropylmalate dehydratase, small subunit  3-isopropylmalate dehydratase, large subunit  3-isopropylmalate dehydrogenase  adenosylcobalamin-dependent diol dehydratase beta subunit  acetolactate synthase, small subunit  acetolactate synthase, large subunit, biosynthetic type  2-isopropylmalate synthase  threonine dehydratase, biosynthetic  dihydroxy-acid dehydratase  homoserine kinase  phosphoribosyl-ATP pyrophosphohydrolase  **Biosynthesis of cofactors, prosthetic groups, and carriers**  precorrin-6y c5,15-methyltransferase, putative  pyrazinamidase/nicotinamidase, putative  ferrochelatase  O-succinylbenzoic acid--CoA ligase, putative  **Cell envelope**  membrane protein  D-alanyl-D-alanine carboxypeptidase  **Cellular processes**  hemolysin  TraC protein  TraC protein  **Central intermediary metabolism**  acetyltransferase, putative  acid phosphatase, putative  hydrolase, haloacid dehalogenase-like family  acid phosphatase, putative  diol dehydrase (diol dehydratase) gamma subunit  **DNA metabolism**  DNA topoisomerase IV, A subunit  DNA topoisomerase IV, B subunit  DNA polymerase III, alpha subunit  recombination protein RecR  **Energy metabolism**  aldehyde-alcohol dehydrogenase  alcohol dehydrogenase, iron-containing  carbon dioxide concentrating mechanism protein  chitinase, putative  enolase  glycerol-3-phosphate dehydrogenase, NAD-dependent  phosphoglycerate mutase, 2,3-bisphosphoglycerate-independent  Phosphofructokinase  (R)-2-hydroxyglutaryl-CoA dehydratase activator-related protein  **Fatty acid and phospholipid metabolism**  ethanolamine utilization protein eutj  3-oxoacyl-(acyl-carrier-protein) synthase II  aldehyde-alcohol dehydrogenase, putative  acetyl-CoA carboxylase, carboxyl transferase, alpha subunit  3-oxoacyl-(acyl-carrier-protein) synthase III  **Protein fate**  peptidase T, putative  peptidase, M16 family  proline dipeptidase  **Protein synthesis**  ribosomal protein L7/L12  ribosomal protein L10  ribosomal protein S20  RNA methyltransferase, TrmH family  ribosomal protein L5  ribosomal protein L29  ribosomal protein L11  ribosomal protein L1  ribosomal protein S5  ribosomal protein S8  leucyl-tRNA synthetase  ribosomal protein L16  ribosomal protein L6  ribosomal protein L24  ribosomal protein S17  ribosomal protein S14p/S29e  ribosomal protein L18  ribosomal protein S3  ribosomal protein L28  ribosomal protein L30  ribosomal protein L14  ribosomal protein L22  ribosomal protein L20  ribosomal protein S15  ribosomal protein L27  SsrA-binding protein  glutamyl-tRNA synthetase  ribosomal protein L17  ribosomal protein S18  ribosomal protein L15  translation elongation factor Tu  ribosomal protein S16  **Purines, pyrimidines, nucleosides, and nucleotides**  phosphoribosylamine--glycine ligase  phosphoribosylglycinamide formyltransferase  phosphoribosylaminoimidazolecarboxamide formyltransferase/IMP cyclohydrolase  phosphoribosylformylglycinamidine cyclo-ligase  phosphoribosylformylglycinamidine synthase II  amidophosphoribosyltransferase  phosphoribosylformylglycinamidine synthetase I  anaerobic ribonucleoside-triphosphate reductase  adenylate kinase  phosphoribosylaminoimidazole-succinocarboxamide synthase  **Regulatory functions**  transcriptional antiterminator, bglG family, putative  Nitrogen regulatory protein P-II  transcriptional regulator, TetR family domain protein  dipeptide transport operon repressor  transcriptional regulator, Fur family  transcriptional regulator, gntR family domain protein  transcriptional regulator, TetR family  **Transcription**  ribosome-binding factor A  ribonuclease R  **Transport and binding proteins**  ABC transporter, ATP-binding protein  PTS system, fructose-specific IIABC component  PTS system, fructose-specific IIBC component  oligopeptide-binding protein appa precursor, putative  glycerol uptake facilitator protein  PTS system, fructose-specific IIBC component  ABC transporter, ATP-binding protein, putative  MATE efflux family protein, putative  peptide ABC transporter, permease protein  xanthine/uracil permease family protein  ABC transporter efflux protein, DrrB family, putative  Na+/H+ antiporter, MnhE component  oligopeptide transport permease protein appc  sugar ABC transporter, permease protein  amino acid permease  ABC transporter, permease protein  Na+/H+ antiporter, MnhF component  osmoprotectant ABC transporter, permease protein  ABC transporter, ATP-binding protein  amino acid ABC transporter, permease protein  Na+/H+ antiporter, MnhD component  cobalt transport protein cbin  ABC transporter, ATP-binding protein  **Hypothetical and unclassified proteins**  pduJ protein  diol dehydrase(diol dehydratase) alpha subunit  PduM, putative  pduJ protein  PduL  pdub protein  PduG  DUF80 domain protein  Conserved membrane protein, possible permease, YHCI B.subtilis ortholog, putative  S4 domain protein  succinate dehydrogenase/fumarate reductase, flavoprotein subunit  YlmG protein  KH domain protein  Phospholipase/Carboxylesterase superfamily  PduH  tributyrin esterase  phosphoribosyl-ATP pyrophosphatase/phosphoribosyl-AMP cyclohydrolase  Uncharacterized protein family (UPF0051) family  GTP-binding protein TypA  GTP-binding protein LepA  conserved hypothetical protein  conserved hypothetical protein  conserved hypothetical protein  conserved hypothetical protein  conserved hypothetical protein TIGR00302  conserved hypothetical protein  conserved hypothetical protein  conserved hypothetical protein  conserved hypothetical protein  conserved hypothetical protein  conserved hypothetical protein  conserved hypothetical protein  conserved hypothetical protein  conserved hypothetical protein  hypothetical protein  hypothetical protein |

^a^ [1]

^b^ The log_2_-transformed mean fold change values were calculated from three biological replicates. The genes listed are those displaying ≥ 2.5 –fold (equivalent to ±1.3 log_2_) change (rounded to one digit) in transcript level between the *∆sigB, ∆sigL* and *∆sigBL* and the parental EGD-e strain with a moderated t-test statistical significance of P-value≤0.01.

^c^ Gene functional categories are based on the annotations provided by the Comprehensive Microbial Resource of the J. Craig Venter Institute (CMR-JCVI) (<http://cmr.jcvi.org>).

References

1. Mattila, M.; Somervuo, P.; Rattei, T.; Korkeala, H.; Stephan, R.; Tasara, T. Phenotypic and transcriptomic analyses of Sigma L−dependent characteristics in Listeria monocytogenes EGD−e. *Food Microbiol.* **2012**, *32*, 152–164
